# A bacterial biosynthetic pathway for methylated furan fatty acids

**DOI:** 10.1074/jbc.RA120.013697

**Published:** 2020-05-20

**Authors:** Rachelle A. S. Lemke, Stephanie M. Olson, Kaitlin Morse, Steven D. Karlen, Alan Higbee, Emily T. Beebe, John Ralph, Joshua J. Coon, Brian G. Fox, Timothy J. Donohue

**Affiliations:** 1 Department of Bacteriology, University of Wisconsin, Madison, Wisconsin, USA; 2 Wisconsin Energy Institute, University of Wisconsin, Madison, Wisconsin, USA; 3 Great Lakes Bioenergy Research Center, University of Wisconsin, Madison, Wisconsin, USA; 4 Department of Biochemistry, University of Wisconsin, Madison, Wisconsin, USA; 5 Genome Center of Wisconsin, University of Wisconsin, Madison, Wisconsin, USA; 6 Department of Biomolecular Chemistry, University of Wisconsin, Madison, Wisconsin, USA

**Keywords:** furan fatty acid, fatty acid metabolism, fatty acyl methylase, fatty acid modification, lipid metabolism, oxygenated fatty acids, phospholipid, polyunsaturated fatty acid, α-proteobacteria, cell signaling, polyunsaturated fatty acid (PUFA)

## Abstract

Fatty acids play many important roles in cells and also in industrial processes. Furan fatty acids (FuFAs) are present in the lipids of some plant, fish, and microbial species and appear to function as second messengers in pathways that protect cells from membrane-damaging agents. We report here the results of chemical, genetic, and synthetic biology experiments to decipher the biosynthesis of the monomethylated FuFA, methyl 9-(3-methyl-5-pentylfuran-2-yl) nonanoate (9M5-FuFA), and its dimethyl counterpart, methyl 9-(3,4-dimethyl-5-pentylfuran-2-yl) nonanoate (9D5-FuFA), in two α-proteobacteria. Each of the steps in FuFA biosynthesis occurs on pre-existing phospholipid fatty acid chains, and we identified pathway intermediates and the gene products that catalyze 9M5-FuFA and 9D5-FuFA synthesis in *Rhodobacter sphaeroides* 2.4.1 and *Rhodopseudomonas palustris* CGA009. One previously unknown pathway intermediate was a methylated diunsaturated fatty acid, (10*E,*12*E*)-11-methyloctadeca-10,12-dienoic acid (11Me-10*t*,12*t*-18:2), produced from (11*E*)-methyloctadeca-11-enoic acid (11Me-12*t*-18:1) by a newly identified fatty acid desaturase, UfaD. We also show that molecular oxygen (O_2_) is the source of the oxygen atom in the furan ring of 9M5-FuFA, and our findings predict that an O_2_-derived oxygen atom is incorporated into 9M5-FuFA via a protein, UfaO, that uses the 11Me-10*t,*12*t*-18:2 fatty acid phospholipid chain as a substrate. We discovered that *R. palustris* also contains a SAM-dependent methylase, FufM, that produces 9D5-FuFA from 9M5-FuFA. These results uncover the biochemical sequence of intermediates in a bacterial pathway for 9M5-FuFA and 9D5-FuFA biosynthesis and suggest the existence of homologs of the enzymes identified here that could function in FuFA biosynthesis in other organisms.

Fatty acids have numerous cellular and biotechnological functions. In biological membranes, fatty acids form and stabilize the hydrophobic component of the bilayer, act as a permeability barrier, influence the activity of integral membrane proteins, and function as secondary messengers in signaling pathways (1–3). Changes in membrane fatty acid composition often help cells maintain viability in response to temperature and environmental changes and protect them from damage caused by many membrane-active agents (2–8). Industrially, fatty acids or compounds derived from them can serve as food additives, antioxidants, anti-inflammatory compounds, lubricants, or substitutes for other compounds that are typically derived from petroleum (9–12). There is significant potential to produce natural or modified fatty acids through synthetic biology or cellular engineering, given the range of known acyl chains and the number of gene products predicted to produce or modify these compounds across the phylogeny. We are studying the biosynthetic pathway for an unusual yet important fatty acid, a 19-carbon monomethylated furan fatty acid, 9-(3-methyl-5-pentylfuran-2-yl)-nonanoic acid, alternatively named 10,13-epoxy-11-methyl-octadecadienoic acid (9M5-FuFA) (13, 14).

9M5-FuFA is one of a chemically diverse set of furan fatty acids (FuFAs) found in the lipids of select plants, fish, and microbes (15). FuFAs containing zero, one, or two methyl groups are implicated as second messengers in pathways that protect cells from the toxic effects of membrane-damaging agents (15–17). 9M5-FuFA rapidly disappears when cells are exposed to singlet oxygen (^1^O_2_), and this reactive oxygen species is bacteriocidal to cells unable to make this FuFA, illustrating the ability of this FuFA to act as an antioxidant and protect cells from the negative effects of toxic compounds (18). The oxygen atom within FuFAs also provides a site for chemical modifications that could increase their industrial value as lubricants, fuel additives, or biofuels (15). However, strategies to produce large quantities of natural or modified FuFAs are limited by a lack of information about their biosynthesis.

An opportunity to dissect the pathway for FuFA biosynthesis was provided by the increased abundance of 9M5-FuFA fatty acyl chains in phospholipids (18) that are found in a mutant of *Rhodobacter sphaeroides* 2.4.1 with increased expression of genes that respond to ^1^O_2_ (5, 19, 20). Previous reports indicate that a ^1^O_2_-inducible protein RSP2144, UfaM, was necessary for 9M5-FuFA synthesis (18, 21). UfaM is a SAM-dependent methylase that synthesizes a methylated *trans*-unsaturated fatty acid, methyl (*E*)-11-methyloctadeca-12-enoate (11Me-12*t*-18:1), from the *cis*-vaccenic acid side chains within phospholipids (Fig. 1). Although 11Me-12*t*-18:1 is a potential intermediate in 9M5-FuFA synthesis, information is lacking on any additional intermediates, the remaining enzyme(s) in this biosynthetic pathway, and other compounds that are needed to produce 9M5-FuFA.

**Figure 1.**
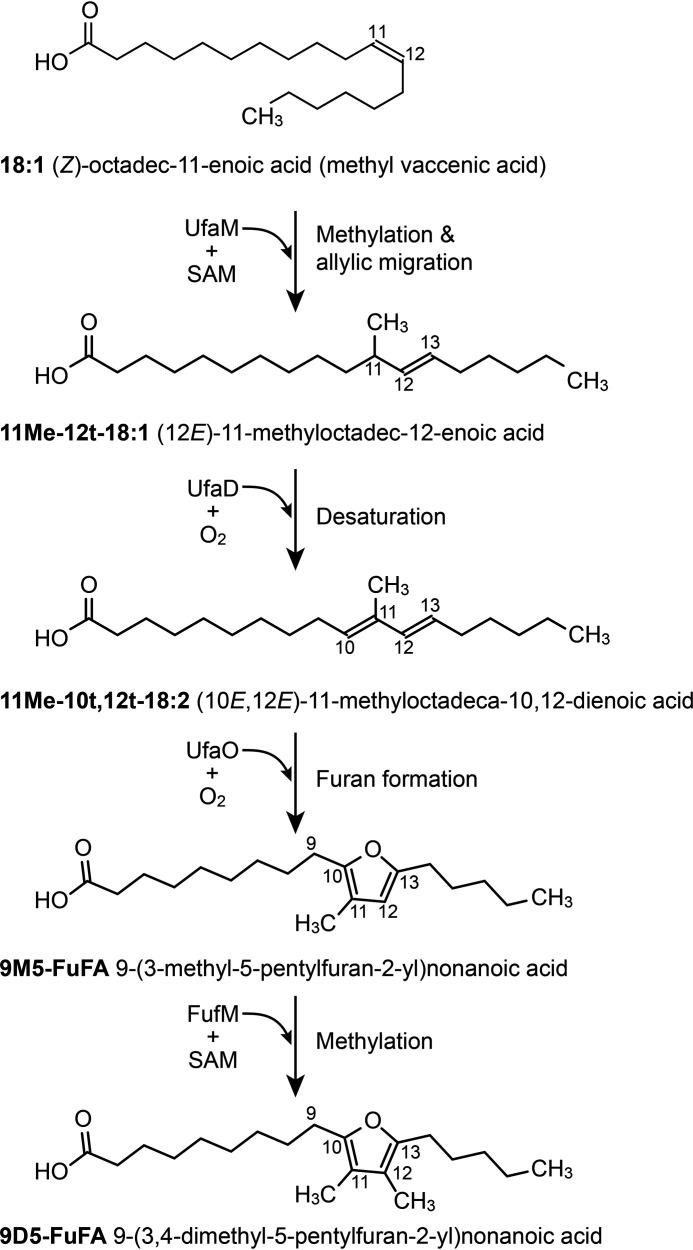
**Furan fatty acids biosynthetic pathway.** Shown is the activity of UfaM as a SAM-dependent methylase that produces 11Me-12*t*-18:1 from vaccenic acid (18:1) (18). In this work, 11Me-12*t*-18:1 is shown to be converted into 11Me-10*t*,12*t*-18:2 in a reaction that is catalyzed by a newly identified fatty acyl desaturase (UfaD). These results also show that 11Me-10*t*,12*t*-18:2 is converted into 9M5-FuFA (Figs. 5 and 6) in an O_2_-dependent reaction catalyzed by UfaO. Finally, 9D5-FuFA is shown to be produced directly from 9M5-FuFA by a (Figs. 7–8) newly identified methylase, FufM (Figs. 9–11).

In this work, a methylated diunsaturated fatty acid, (10*E*,12*E*)-11-methyloctadeca-10,12-dienoic acid (11Me-10*t*,12*t*-18:2), is identified as another intermediate in 9M5-FuFA biosynthesis. Also identified are previously unknown fatty acid–modifying enzymes from two α-proteobacteria (*Rhodobacter sphaeroides* 2.4.1 and *Rhodopseudomonas palustris* CGA009) that synthesize 11Me-10*t,*12*t*-18:2 from 11Me-12*t*-18:1 and convert the methylated diunsaturated fatty acid into 9M5-FuFA (Fig. 1). This work also shows that atmospheric oxygen (O_2_) is the source of the oxygen atom in the furan ring of 9M5-FuFA. We further show that *R. palustris* produces a methyl 9-(3,4-dimethyl-5-pentylfuran-2-yl) (9D5-FuFA), which is the first published report of the synthesis of this fatty acyl chain in bacteria, and that a newly discovered protein acts as a SAM-dependent fatty acid methylase to synthesize diunsaturated 9D5-FuFA from the monounsaturated 9M5-FuFA (Fig. 1). These studies provide important new insights into the biosynthesis of furan rings and information to predict the presence of similar biosynthetic pathways and identify genes that can be used to engineer increased production of FuFAs in prokaryotes and eukaryotes.

## Results

### Genes needed for 9M5-FuFA synthesis

**Figure 2.**
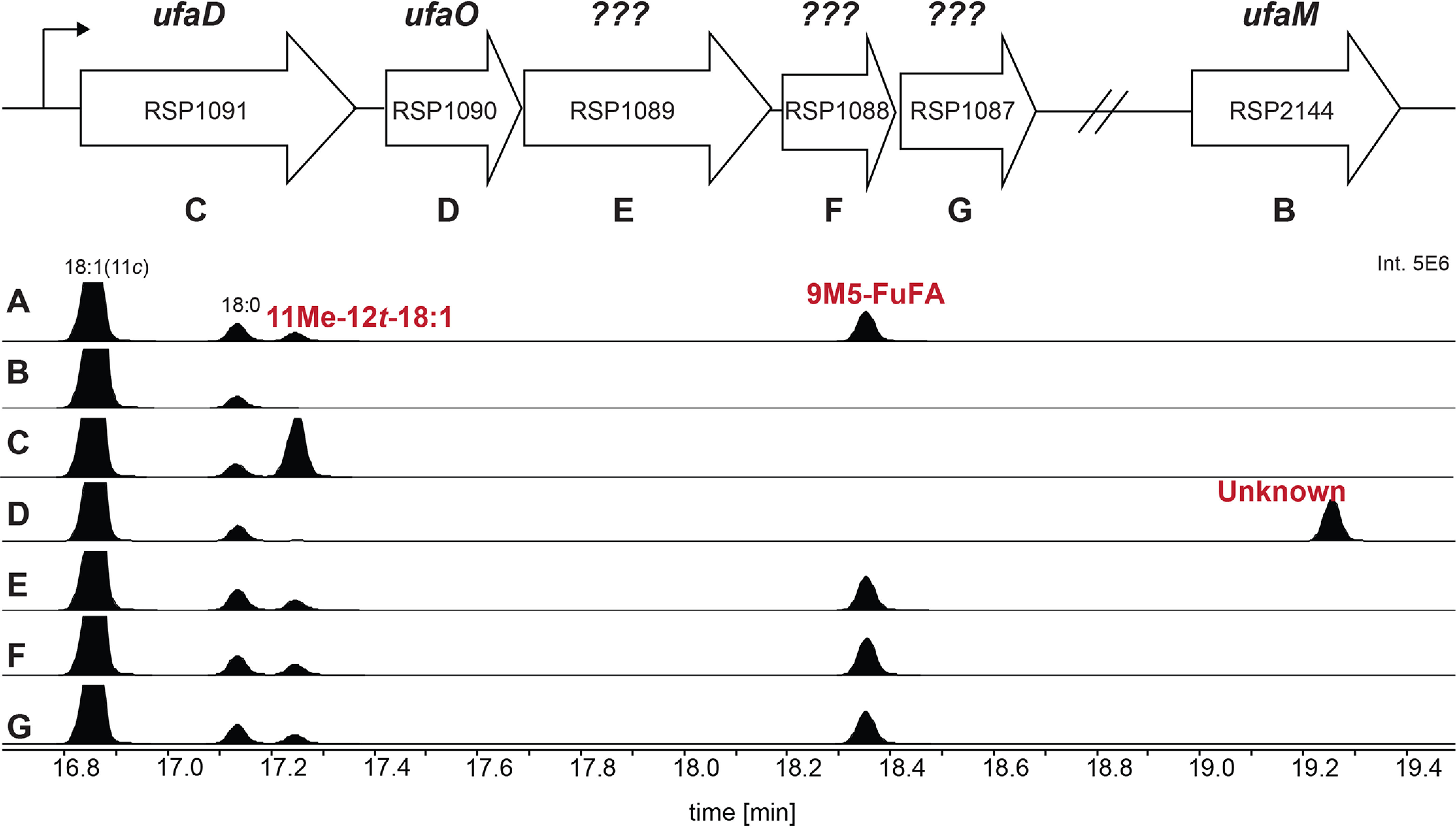
**The conversion of 11Me-12*t*-18:1 into 9M5-FuFA requires both RSP1090 (UfaO) and RSP1091 (UfaD).** *Above* the *panels* is a representation of the genes within the *R. sphaeroides RSP1091-1087* operon. *A–G*, the GC elution profiles of FAMEs generated from Δ*chrR* cells (*A*) as well as Δ*chrR* cells that also contain a *ufa*M deletion (*B*) or contain additional in-frame deletions in *ufaD* (*RSP1091)* (*C*), *ufaO* (*RSP1090*) (*D*), *RSP1089* (*E*), *RSP1088* (*F*), and *RSP1087* (*G*).

Deciphering the FuFA biosynthetic pathway is aided by the knowledge of genes needed for the production of 9M5-FuFA in *R. sphaeroides* (18). For example, fatty acid methyl esters (FAMEs) prepared from the phospholipids found in *R. sphaeroides* Δ*chrR* cells contain higher detectable levels of 9M5-FuFA and 11Me-12*t*-18:1 than WT cells, in addition to the 18:1, 18:0, 16:1, and 16:0 fatty acid chains found in WT cells (Fig. 2*A*). Previous work (18) also showed that loss of UfaM, which catalyzes the SAM-dependent methylation of *cis*-vaccenate (18:1), blocked the accumulation of both 9M5-FuFA and 11Me-12*t*-18:1 (Fig. 2*B*). In addition, a mutation that deleted all five genes in the *RSP1087-1091* operon prevented the accumulation of 9M5-FuFA and led to increased production of 11Me-12*t*-18:1 (18). From these observations, 11Me-12*t*-18:1 was proposed to be an intermediate in 9M5-FuFA synthesis, and one or more products of the RSP1087-1091 operon were potential catalysts of subsequent reactions in 9M5-FuFA synthesis (18).

To test these proposals, the fatty acid composition of phospholipids from mutants lacking both *chrR* (which leads to higher levels of 9M5-FuFA accumulation than in WT cells) and single genes in the *RSP1087-1091* operon were analyzed. Examination of this set of isogenic strains showed that placing individual in-frame deletions of either *RSP1087*, *RSP1088*, or *RSP1089* in Δ*chrR* cells had no detectable impact on the fatty acid profile compared with cells lacking only *chrR* (Fig. 2, *E–G*). This demonstrates that each of these three gene products were not necessary for synthesis of either 9M5-FuFA or 11Me-12*t*-18:1.

In contrast, inactivation of either *RSP1091* (*ufaD*) or *RSP1090* (*ufaO*) in Δ*chrR* cells did alter the fatty acid profile compared with that found in cells lacking only the *chrR* gene (Fig. 2, *C* and *D*). Loss of *ufaD* led to the disappearance of both fatty acids 9M5-FuFA and the previously identified 11Me-12*t*-18:1 (Fig. 2*C*). Δ*chrR* cells lacking *ufaO* were missing fatty acids corresponding to both 9M5-FuFA and 11Me-12*t*-18:1 but contained a previously undetected fatty acid of unknown identity (Fig. 2*D*). These data predict that both *ufaD* and *ufaO* are involved in conversion of 11Me-12*t*18:1 into 9M-FuFA. One possibility is that UfaD converts the previously identified 11Me-12*t*-18:1 into the fatty acid that is derivatized to the unknown FAME and that UfaO catalyzes the conversion of this compound into the furan-containing fatty acid, 9M5-FuFA (Fig. 1).

**Figure 3.**
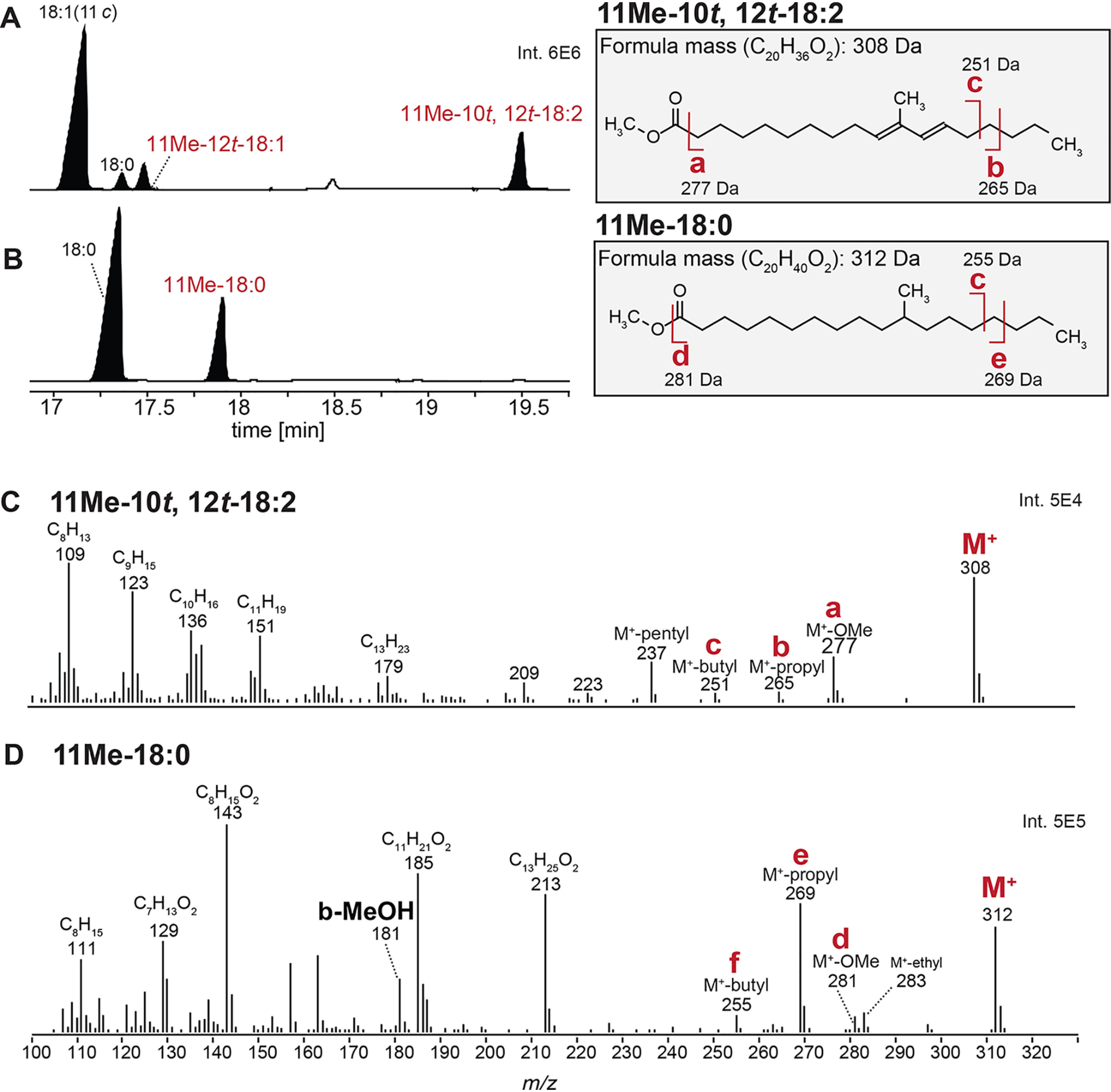
**GC-MS of FAMEs prior to and after hydrogenation.** *A*, the GC profile of FAMEs generated from Δ*chrR*Δ*ufaO* cells before (*A*) and after (*B*) hydrogenation. *C* and *D*, MS fragmentation profiles of FAMEs generated from Δ*chrR*Δ*ufaO* cells before (*C*) and after (*D*) hydrogenation. Fragments *a*–*f* are highlighted in each spectrum as they are diagnostic for the changes in the mass and fragmentation of the molecule after hydrogenation.

To test this hypothesis, the chemical identity of the unknown fatty acid that accumulates in cells lacking *ufaO* needed to be determined. MS analysis of the FAME derivative indicates that it has a molecular mass of 308.2 Da, but analysis of its fragmentation pattern was insufficient to unambiguously elucidate its chemical structure. Hydrogenation of the lipids from cells lacking *ufaO* led to a shift in GC retention time of this FAME (Fig. 3, *A* and *B*) and an increase in the parent ion mass by 4 Da to 312.2 Da (Fig. 3, *C* and *D*). The magnitude of the mass increase after hydrogenation indicates that the untreated FAME contains two double bonds. The new GC retention time and mass were indistinguishable from those of the hydrogenated form of an 11Me-12*t*-18:1 FAME (Fig. 3*B*). These observations suggest that the unknown fatty acid is a diunsaturated derivative of 11Me-12*t*-18:1.

**Figure 4.**
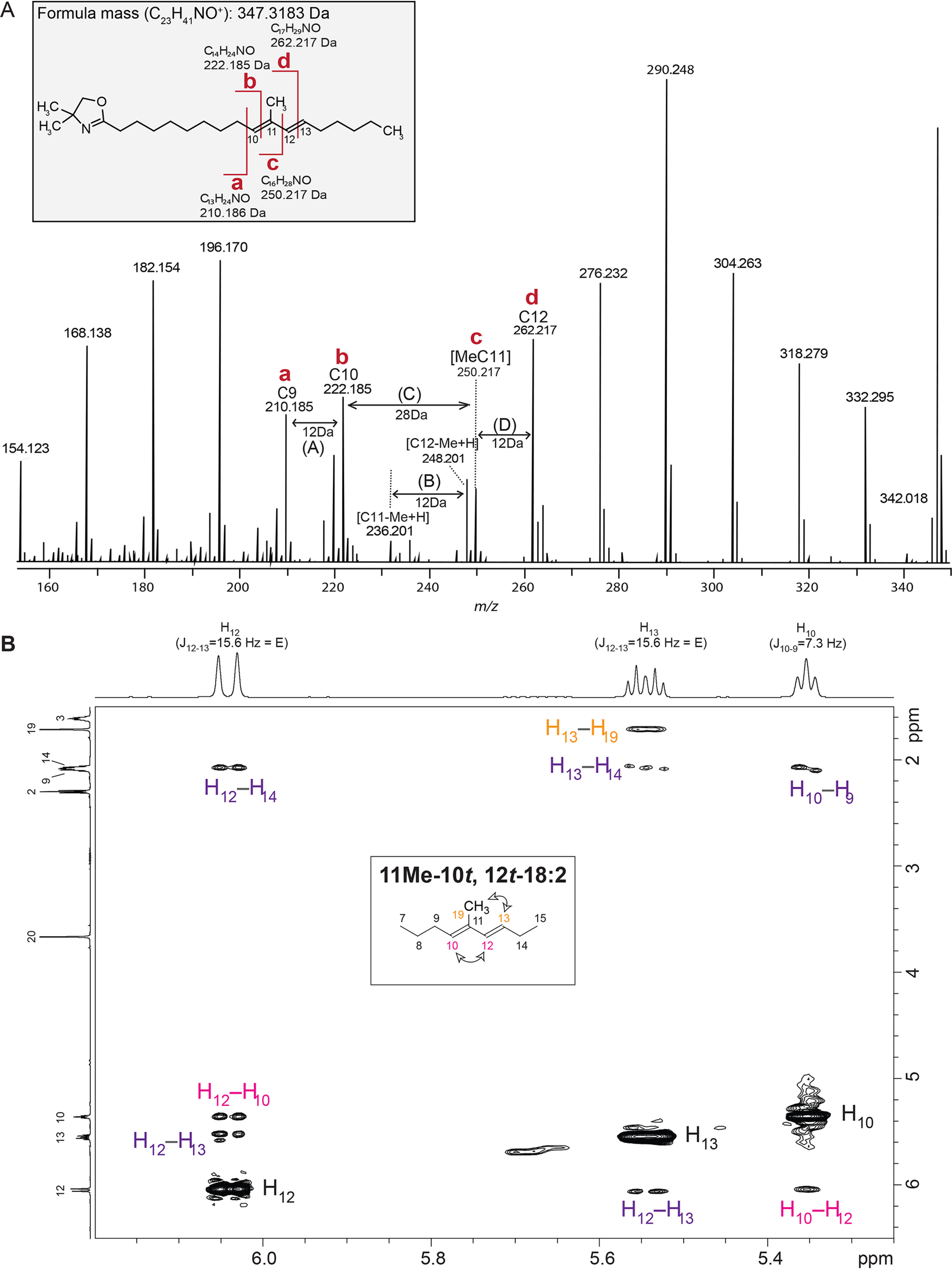
**Identification of the unknown 11Me-10*t*,12*t*-18:2 diunsaturated fatty acid that accumulates in Δ*chrR*Δ*ufaO* cells.** *A*, MS fragmentation of a DMOX derivative of the fatty acid that is accumulated in Δ*chrR*Δ*ufaO* cells. The diagnostic fragmentation products that identify this as a DMOX derivative of an 11Me-10*t*,12*t*-18:2 fatty acid are labeled *a–d* on the structure and mass spectrum. *B*, 2D NOE ^1^H–^1^H correlation spectra of the FAME. The NOE (through-space) correlation peaks indicate the presence of one dominant geometrical isomer (10*t*,12*t*), as was also deduced from GC-MS.

To determine the chemical identity of this unknown fatty acid, several additional experiments were conducted. A 4,4-dimethyloxazoline (DMOX) derivative of phospholipids from cells lacking *ufaO* identified a product whose molecular mass (347.3182 Da) predicted the chemical formula (C_23_H_41_O_1_N_1_), and the fragmentation pattern of the DMOX-modified unknown compound provided additional support for the assignment of the parent fatty acid as a diunsaturated derivative of the monounsaturated 11Me-12*t*-18:1 (Fig. 4*A*). As an independent assessment of this assignment, the two-dimensional NOESY NMR spectrum of purified samples of FAME derivatives of the unknown fatty acid was performed. This analysis identified NMR signals diagnostic of the conjugated double bonds predicted from hydrogenation of the FAME and the MS fingerprints of the DMOX derivative of this compound (Fig. 4). Analysis of the NMR data also indicated that about 98% of the FAME had both double bonds in the *trans*-configuration. Previous studies concluded the double bond in 11Me-12*t*-18:1 was in the *trans*-configuration (22). As such, it appears 11Me-10*t*,*12t*-18:2 differs from 11Me-12*t*-18:1 by the presence of a second *trans* double bond adjacent to the methyl group at position 11 in the acyl chain.

### Conversion of 11Me-12t-18:1 to 11Me-10t,12t-18:2 and 9M5-FuFA

**Figure 5.**
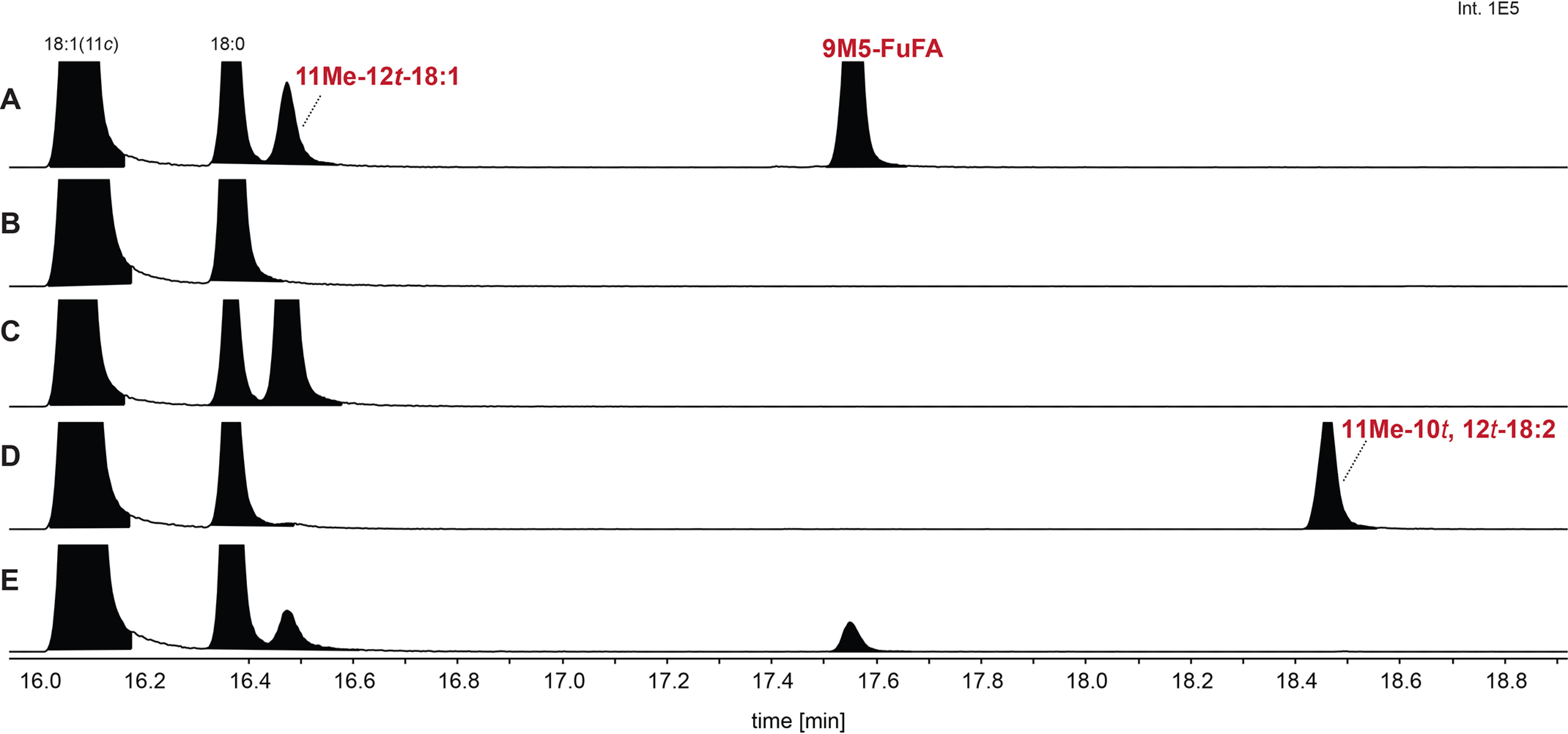
**FAME profiles produced from *R. sphaeroides* cells ectopically expressing *R. sphaeroides* 9M5-FuFA biosynthetic genes.** Shown are GC elution profiles of FAMEs prepared from *R. sphaeroides* Δ*chrR* cells (*A*) or Δ*ufaM*Δ*ufaD*Δ*ufaO* cells lacking *ufa*M (*B*), containing *ufa*M (*C*), containing *ufa*M and *ufaD* (*D*), or containing the combination of *ufa*M, *ufaD*, and *ufaO* (*E*) on a plasmid under control of an IPTG-inducible promoter.

The above results predict that the FuFA biosynthetic pathway utilizes three gene products (*ufaM*, *ufaD*, and *ufaO*) that sequentially convert *cis*-vaccinate (18:1(11*E*)) into 11Me-12*t*-18:1, 11Me-10*t*,12*t*-18:2, and 9M5-FuFA (Fig. 1). To test this hypothesis, FAME profiles of phospholipids prepared from cells containing different combinations of *ufaM*, *ufaD*, and *ufaO* cloned behind an isopropyl β-d-1-thiogalactopyranoside (IPTG)-inducible promoter on a low-copy plasmid were examined. The parent strain for this study contained an intact *chrR* gene but lacked all three of these genes (*ufaM*, *ufaD*, and *ufaO*) on the chromosome. Use of this strain allowed us to separate the synthesis of 9M5-FuFA and its putative biosynthetic intermediates from the increases in expression of other genes that are caused by the loss of *chrR* (Fig. 5*A*) (23, 24). FAMEs of phospholipids from the parent strain (missing *ufaM*, *ufaD*, and *ufaO* on the chromosome) lacked detectable levels of 9M5-FuFA or any of the putative FuFA pathway intermediates (Fig. 5*B*). In contrast, when this parent strain contains only the *ufaM* gene on a plasmid, it accumulated a fatty acid corresponding to 11Me-12*t*-18:1 (Fig. 5*C*), as predicted from its known activity as a fatty acyl methylase (18). In addition, fatty acids isolated from this parent strain containing *ufaM* plus *ufaD* on the same plasmid produced 11Me-10*t*,12*t*-18:2 (Fig. 5*D*), whereas fatty acids isolated from the same strain containing *ufaM* plus *ufaD* and *ufaO* on this plasmid accumulated 9M5-FuFA (Fig. 5*D*). These results predict that these three genes are sufficient *in vivo* for 9M5-FuFA synthesis from *cis*-vaccenic acid (18:1), the fatty acid substrate for the SAM-dependent methylase, UfaM (18).

### 11Me-10t,12t-18:2 is a product of desaturation of 11Me-12t-18:1

**Figure 6.**
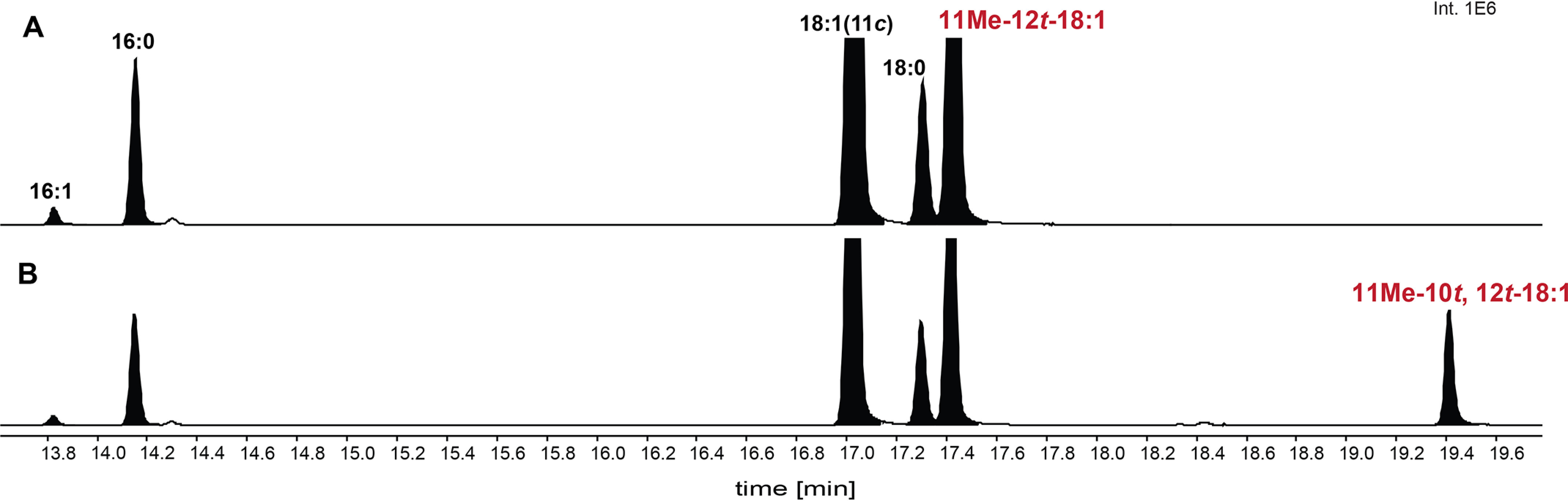
**Analysis of FAMEs produced from *R. sphaeroides* Δ*chrR*Δ*ufaD* phospholipids before (*A*) and after (*B*) incubation with recombinant His_8_-UfaD protein.** Shown are GC elution profiles of FAMEs from phospholipid liposomes prepared from *R. sphaeroides* Δ*chrR*Δ*ufaD* cells incubated in the absence (*A*) or presence (*B*) of recombinant His_8_-UfaD protein.

The results of the above experiments further predict that 11Me-10*t*,12*t*-18:2 is converted from 11Me-12*t*-18:1 by UfaD. To test this hypothesis, a recombinant His_8_-tagged version of UfaD was incubated with *R. sphaeroides* phospholipid-derived liposomes and tested for fatty acyl modifications. When assays were performed in the presence of liposomes that contained 11Me-12*t*-18:1 fatty acids (prepared from a strain lacking *ufaD*), the predicted fatty acid substrate for desaturation, a FAME with a GC retention time and MS fragmentation pattern identical to methyl 11Me-10*t*,12*t*-18:2 was detected (Fig. 6, *A* and *B*). All of the detected 18:2 fatty acid products were methylated, suggesting that UfaD did not desaturate the abundant 18:1 fatty acid found in *R. sphaeroides* phospholipids. The results of this experiment allow us to contend that UfaD is a previously unknown fatty acyl desaturase and that this protein has a high degree of specificity for 11Me-12*t*-18:1 as a substrate.

The predicted product of UfaD activity, 11Me-10*t*,12*t*-18:2, accumulates when Δ*chrR* cells also lacking *ufaO* are grown in the presence of O_2_ (Fig. 2*D*). However, 11Me-10*t*,12*t*-18:2 does not accumulate to detectable levels when the same strain was grown in the absence of O_2_ (Fig. S1). Thus, O_2_ is required for 11Me-10*t*,12*t*-18:2 formation, possibly because it serves as an electron acceptor for the newly identified UfaD desaturase (25).

### O_2_ is the source of the oxygen atom in the furan ring of 9M5-FuFA

**Figure 7.**
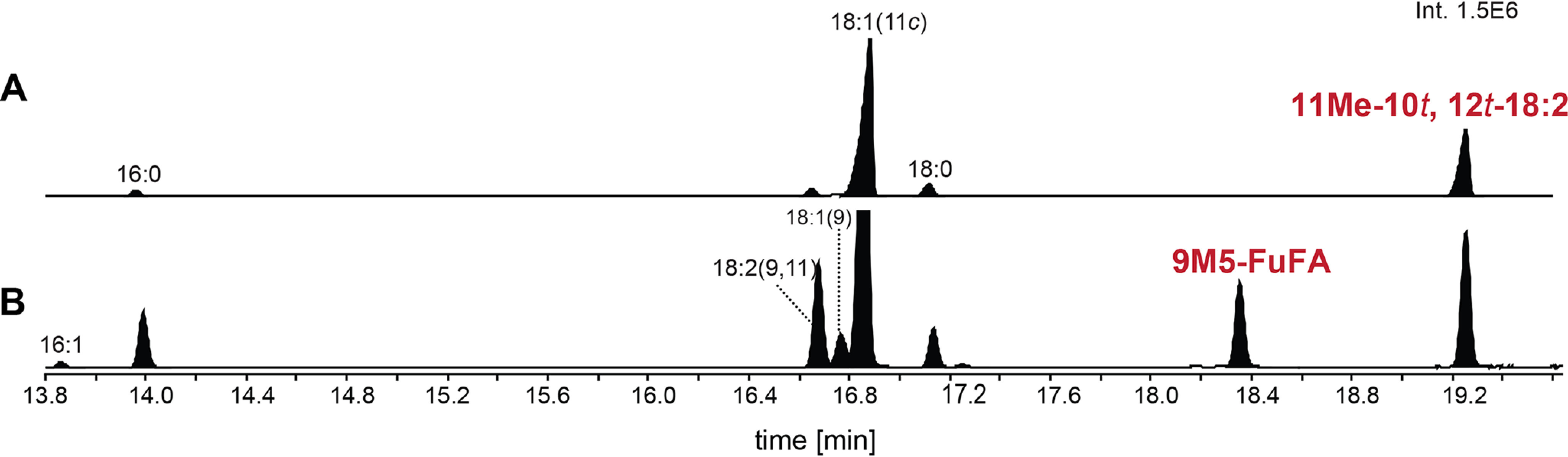
**Analysis of FAMEs produced from *R. sphaeroides* Δ*chrR*Δ*ufaO* phospholipids before and after incubation with recombinant His_6_-UfaO protein.** *A*, GC elution profiles of FAMEs from Δ*chrR*Δ*ufaO* phospholipid liposomes. *B*, FAMEs derived from incubating recombinant His_6_-UfaO with phospholipid liposomes prepared from Δ*chrR*Δ*ufaO* cells.

Another prediction from the above data are that 9M5-FuFA is derived from 11Me-10*t*,12*t-*18:2. When a recombinant His_6_-UfaO protein synthesized *in vitro* using a wheat germ cell-free extract was incubated with *R. sphaeroides* liposomes containing 11Me-10*t*,12*t*-18:2 (a possible substrate for UfaO), a FAME with a GC retention time and MS fragmentation pattern indistinguishable from 9M5-FuFA (Fig. 7) was observed.

**Figure 8.**
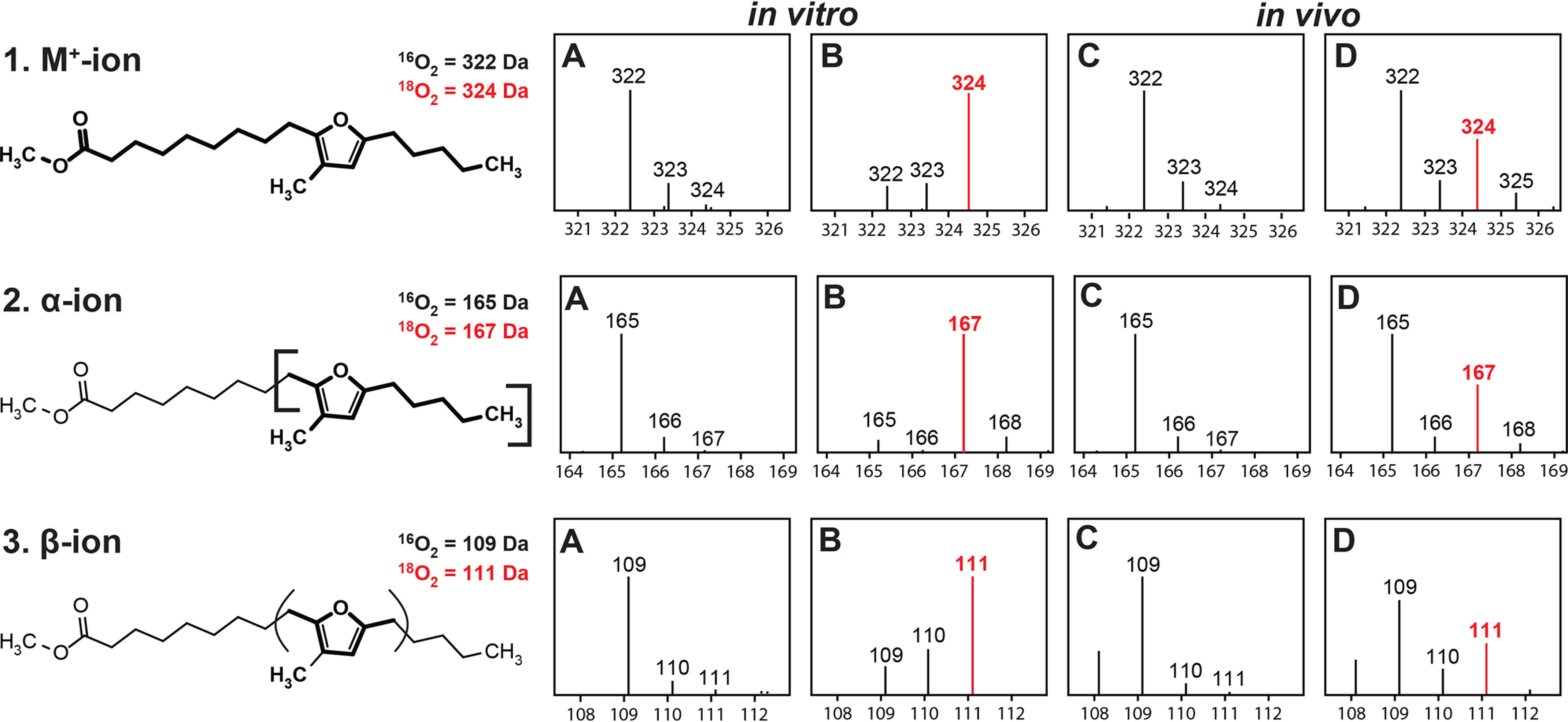
**Mass spectra of 9M5-FuFA produced in the presence of either ^16^O_2_ or ^18^O_2_.** *A* and *B*, MS fragmentation patterns of key ions (M^+^, α, and β) from the fatty acid produced by recombinant His_6_-UfaO (Fig. 7) in a reaction performed in the presence of natural abundance O_2_ (*A*) or ^18^O_2_ (*B*) *in vitro*. *C* and *D*, MS fragmentation pattern of the fatty acid that is produced *in vivo* when Δ*chrR* cells are grown in the presence of natural abundance O_2_ (*C*) or ^18^O_2_ (*D*).

To test the source of the oxygen atom in the furan ring of 9M5-FuFA, this assay was repeated under an atmosphere of ^18^O_2_ (99% isotopic enrichment). In these reaction products, the diagnostic M^+^ (324.3 Da), α-ion (167.1 Da), and β-ion (111.1 Da) derived from 9M5-FuFA each had a mass that was ∼2 Da larger than the ones present when the reaction was performed in the presence of air (322.2, 165.1, and 109.1 Da, respectively, Fig. 8 (*A* and *B*)). The observed 2-Da increase in the mass of these ions and their abundance compared with their counterparts when a control reaction was performed in ^16^O_2_ atmosphere shows that O_2_ is the source of the oxygen atom in the furan ring of 9M5-FuFA.

As an independent test of the source of the oxygen atom in 9M5-FuFA, the fatty acid profile of Δ*chrR* cells grown aerobically in the presence of either ^16^O_2_ (Fig. 8*C*) or ^18^O_2_ (Fig. 8*D*) were analyzed. When cells are grown in the presence of ^18^O_2_, the diagnostic M^+^, α-ion, and β-ion from 9M5-FuFA FAMEs were 2 Da larger than those found when the same strain is grown in the presence of air. In addition, FAMEs derived from the 9M5-FuFA obtained from cells grown in the presence of ^18^O_2_ have a mass and fragmentation pattern indistinguishable from the product of UfaO activity (Fig. 8*B*).

Based on the results of the above *in vitro* and *in vivo* experiments, O_2_ is the direct source of the oxygen atom in the furan ring of 9M5-FuFA, suggesting that UfaO is a previously unknown fatty acyl–modifying enzyme that uses the methylated diunsaturated fatty acid, 11Me-10*t*,12*t*-18:2, as a substrate. The use of O_2_ as the source of the oxygen atom in 9M5-FuFA is consistent with the results of previous *in vivo* experiments that demonstrated that this FuFA only accumulated in phospholipids when Δ*chrR* cells were grown under aerobic conditions (18).

### The FuFA biosynthetic pathway exists in other bacteria

**Figure 9.**
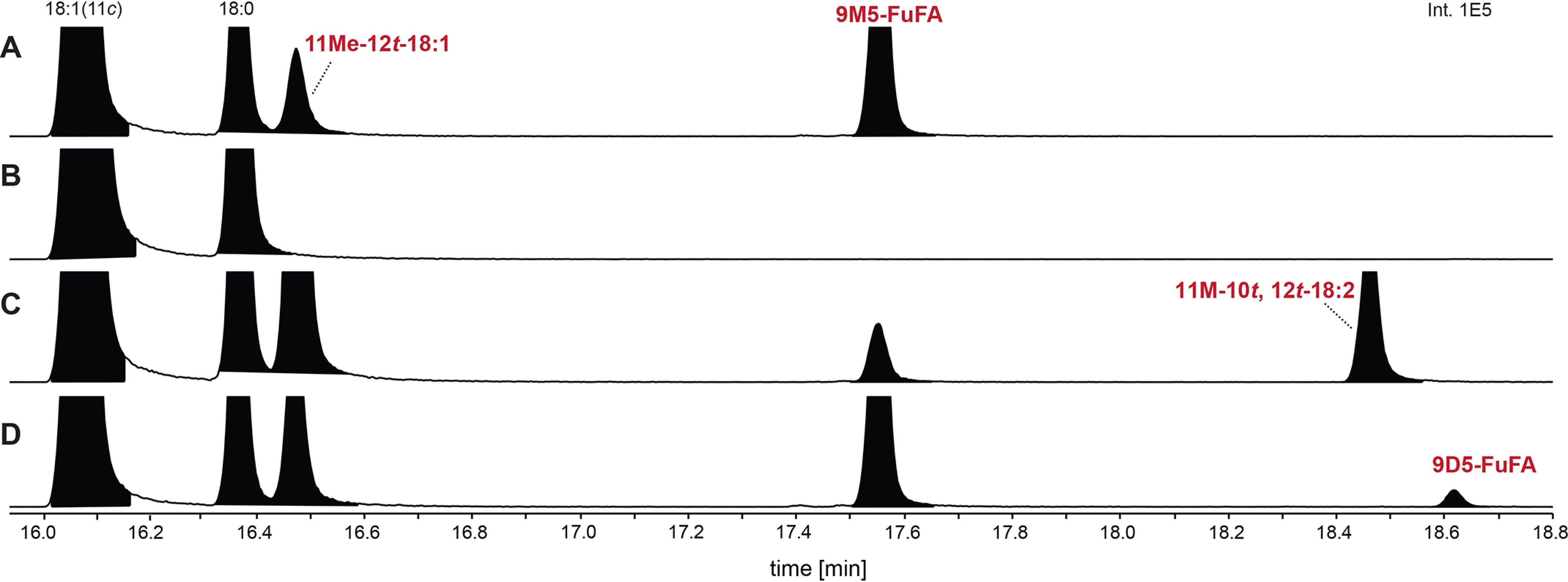
**The FAME profiles produced from *R. sphaeroides* cells ectopically expressing *R. palustris* 9D5-FuFA synthesis genes.** *A* and *B*, GC elution profiles of the FAMEs prepared from *R. sphaeroides* Δ*chrR* cells, biological replicate of Fig. 5*A* (*A*), and Δ*ufaM*Δ*ufaD*Δ*ufaO* cells, biological replicate of Fig. 5*B* (*B*). *C*, GC elution profiles of FAMEs produced from *R. sphaeroides* Δ*ufaM*Δ*ufaD*Δ*ufaO* cells expressing the *R. palustris RPA2569*–*2571* genes on a plasmid with an IPTG-inducible promoter. *D*, FAME profile produced from *R. sphaeroides* Δ*chrR* cells expressing *fufM*.

Other cells are predicted to contain proteins related to UfaM, UfaD, and UfaO (24). To test this hypothesis, we tested whether the *R. palustris* UfaM, UfaD, and UfaO homologs (*RPA2569*, *RPA2571,* and *RPA2570*, respectively) were sufficient to produce the expected fatty acyl chains. To do this, these three genes were cloned behind an IPTG-inducible promoter on a low-copy plasmid and placed in the *R. sphaeroides* Δ*ufaM*, Δ*ufaD*, and Δ*ufaO* triple-mutant strain. Analysis of FAMEs prepared from phospholipids from this strain showed that these three *R. palustris* genes are sufficient to produce 11Me-12*t*-18:1, 11Me-10*t*,12*t*-18:2, and 9M5-FuFA in *R. sphaeroides* (Fig. 9*C*) (Fig. 9 (*A* and *B*) shows data from biological replicates of Fig. 5 (*A* and *B*)). These data demonstrate that proteins homologous to *R. sphaeroides* UfaM, UfaD, and UfaO are sufficient for production of 9M5-FuFA and its predicted pathway intermediates.

**Figure 10.**
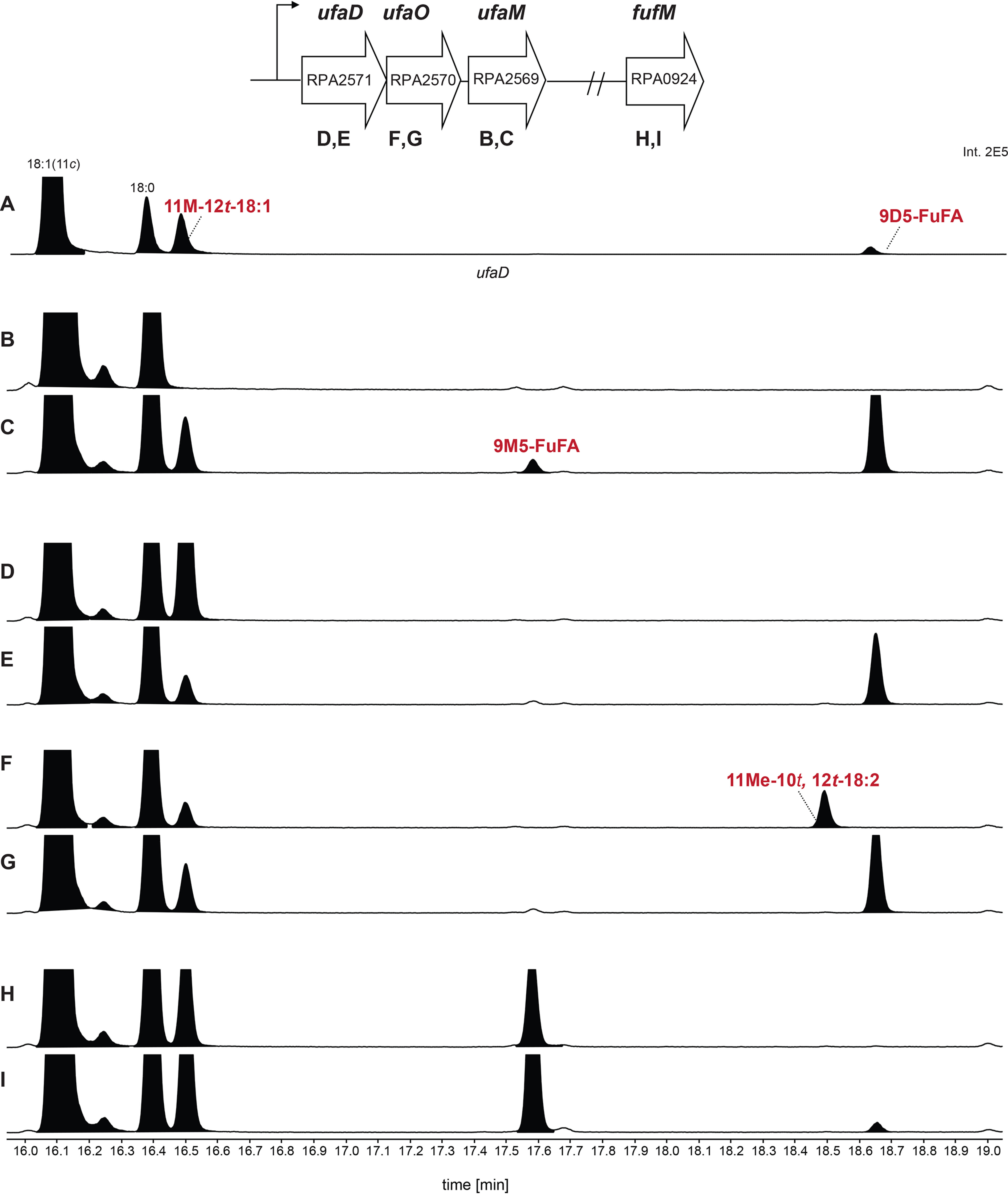
**The FAME profiles produced from *R. palustris* CGA009 and mutant strains.** *A–I*, GC elution profiles of the FAMEs prepared from the indicated *R. palustris* strains containing an in-frame deletion of individual *R. palustris* genes without or with the noted gene on a plasmid. *A*, WT CGA009; *B*, Δ*RPA2569*; *C*, ΔRPA*2569* + *ufaM*; *D*, Δ*RPA2571*; *E*, Δ*RPA 2571* + *ufaD*; *F*, Δ*RPA2570*; *G*, Δ*RPA2570* + *ufaO*; *H*, Δ*RPA0924*; *I*, Δ*RPA0924* + *FufM*.

The above finding led us to ask whether *R. palustris* contained intermediates or products of the 9M5-FuFA pathway. It was especially important to test this prediction as published analyses do not report the accumulation of a FuFA (9M5-FuFA or others) in *R. palustris* (26). By analysis of the FAME profile of phospholipids from aerobically grown *R. palustris* cells, we identified 9M5-FuFA and 11Me-12*t*-18:1 (Fig. 10). The accumulation of these compounds was abolished when cells contained an in-frame deletion of *RPA2569* (*ufaM*), *RPA2570* (*ufaO*), and *RPA2571*(*ufaD*), but when these three genes were expressed from a plasmid in the Δ*RPA2569–2571* mutant, 11Me-12*t*-18:1 and 9M5-FuFA production was restored (Fig. 10). From these results, we conclude that the RPA2569–2571 gene products are necessary and sufficient for 9M5-FuFA production by *R. palustris*.

### Bacterial synthesis of a dimethyl-FuFA

**Figure 11.**
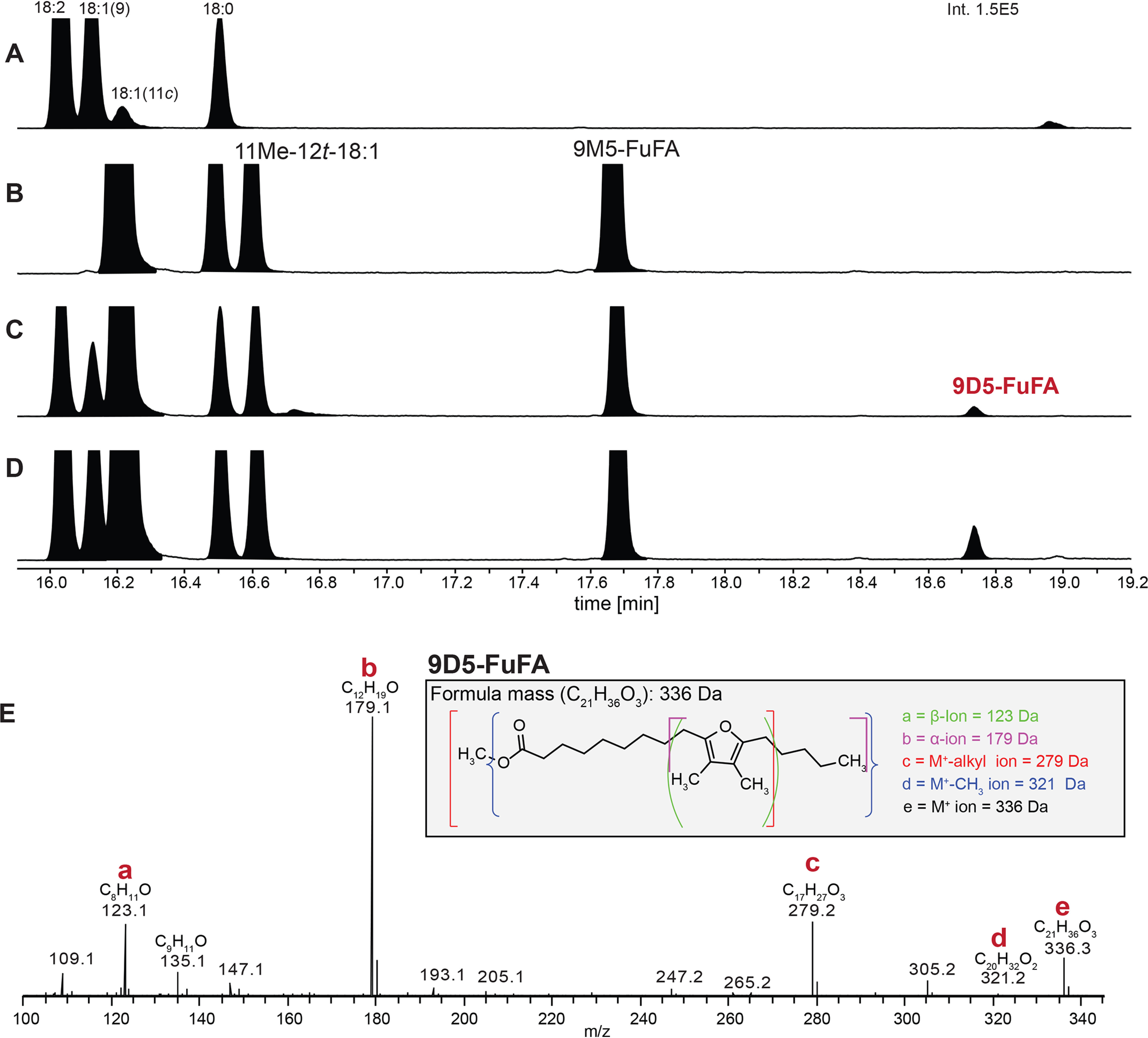
**Analysis of FAMEs produced from *in vitro* incubation of phospholipids with His_6_-FufM protein.** *A*, GC elution profiles of FAMEs from lipids in wheat germ cell-free extract. *B*, FAMEs from phospholipid liposomes prepared from Δ*chrR* cells. *C*, FAMEs in which phospholipid liposomes from Δ*chrR* cells were mixed with His_6_-FufM protein synthesized using wheat germ cell-free extract. *D*, FAMEs after the materials in *C* were incubated with 25 μm SAM. *E*, mass spectrum of the FAME that elutes at 18.75 min with key diagnostic ions for 9D5-FuFA *highlighted*.

The above analysis of *R. palustris* phospholipid FAMEs showed that this bacterium contained a product with a molecular mass 336.52 and a MS fragmentation pattern (Figs. 10 and 11) diagnostic of a dimethyl-FuFA, 9D5-FuFA (Fig. 1). Accumulation of the putative 9D5-FuFA is abolished when the *RPA2569–2571* genes are inactivated (Fig. 10), suggesting that this compound is synthesized from 9M5-FuFA via a heretofore unidentified FuFA methylase. The *R. palustris* genome contains two genes annotated as SAM-dependent fatty acid–modifying enzymes (*RPA3082* and *RPA0924*) with 38 and 26% amino acid identity, respectively, to the known *R. sphaeroides* fatty acyl methylase UfaM (26).

To test the role of these putative fatty acid–modifying enzymes in the synthesis of 9D5-FuFA, we analyzed the impact of inactivating each of these genes on the phospholipid profile. There were no significant changes in the profile when *RPA3082* was deleted (Fig. S2). In contrast, when cells lacked only the *RPA0924* (*fufM*) gene, 9M5-FuFA was the only FuFA produced, implicating this protein in synthesis of 9D5-FuFA. To test this hypothesis, *fufM* was cloned behind an IPTG-inducible promoter and expressed in *R. sphaeroides* Δ*chrR* cells that accumulate 9M5-FuFA (Fig. 2). This *R. sphaeroides* strain accumulated 9D5-FuFA (Fig. 9*D*). Thus, we predict that *fufM* is sufficient for 9D5-FuFA synthesis, presumably because it methylates 9M5-FuFA. Indeed, when we incubated an *in vitro* synthesized FufM protein with SAM and 9M5-FuFA-containing liposomes (prepared from *R. sphaeroides* Δ*chrR* cells), there was synthesis of a compound with a retention time and MS spectrum indistinguishable from the 9D5-FuFA FAME (Fig. 11). Based on this, FufM is a previously uncharacterized 9M5-FuFA methylase that produces 9D5-FuFA.

## Discussion

FuFAs are a broad class of fatty acids with zero, one, or two methyl groups in oxygen-containing (epoxy) rings that are positioned at different places in the acyl chain (13, 15). This work describes a previously unknown bacterial pathway for the synthesis of mono- and dimethyl-FuFAs, a poorly characterized group of membrane fatty acids found in microbes, animals, and plants. Despite the known or predicted roles of FuFAs as bilayer components or cellular antioxidants (15), little is known about their biosynthesis (22, 27). Our study identified additional intermediates in FuFA biosynthesis, reports on several previously unknown enzymes involved in mono- and dimethyl-FuFA biosynthesis, and demonstrates a role for O_2_ in two steps of this pathway. Below, we relate our findings to previous analyses of bacterial FuFA synthesis, describe the chemistry involved in biosynthesis of these fatty acids, and illustrate how our findings can be used to predict the existence of similar pathways in other biological systems.

### The FuFA biosynthetic pathway

Previous work showed that *R. sphaeroides* synthesized 9M5-FuFA by modifying pre-existing phospholipid fatty acyl chains (18). Several biosynthetic schemes have been proposed for mono- or dimethyl-FuFA synthesis in prokaryotes and eukaryotes, each of which assumes that these acyl chains are derived from pre-existing fatty acids (13, 14, 22, 27, 28). However, there is no experimental evidence for predicted pathway intermediates, the identity of FuFA biosynthetic enzymes, the source of oxygen in the furan ring, or whether dimethyl-FuFA is synthesized directly from monomethylated derivatives.

The only biochemical data on intermediates in FuFA biosynthesis come from studies with *R. sphaeroides*. In this bacterium, the first step of 9M5-FuFA synthesis is catalyzed by a SAM-dependent fatty acyl methylase (UfaM) that produces a methylated unsaturated fatty acid (11Me-12*t*-18:1) from *cis*-vaccenic acid (18). However, it is not known whether 11Me-12*t*-18:1 directly converts to 9M5-FuFA or if additional intermediates are involved in the biosynthesis of this fatty acid chain; nor are the identity and biochemical properties of other enzymes needed to synthesize this FuFA known. Below, we summarize new insights gained into FuFA biosynthesis by our studies.

### A newly identified diunsaturated fatty acid intermediate involved in 9M5-FuFA synthesis

The accumulation of a new fatty acid when *ufaO* was inactivated suggested that this compound was a potential intermediate in FuFA biosynthesis. However, the identity of the FAME derivative of this fatty acid could not be unambiguously determined by its mass and fragmentation pattern. Instead, GC-MS of a DMOX derivative and NMR analysis of the FAME was used to identify the unknown fatty acyl chain that accumulates when *ufaO* was inactivated as 11Me-10*t*,12*t*-18:2. The DMOX derivative provides a way to distinguish 11Me-10*t*,12*t*-18:2 from 2-octylcyclopropen-1-octanoic acid whose FAME mass spectrum is identical to that of the diunsaturated FAME. Our data show that UfaD is necessary and sufficient for the synthesis of 11Me-10*t*,12*t*-18:2 from 11Me-12*t*-18:1 and that the newly incorporated double bond in this diunsaturated fatty acid is primarily in the *trans*-configuration.

UfaD is annotated as a fatty acid–modifying enzyme and is predicted to contain an NAD-FAD–binding domain, which has significant amino acid identity to reductases and dehydrogenases (including phytoene dehydrogenases and desaturases) that generate a polyunsaturated intermediate during carotenoid biosynthesis) (29). As 11Me-10*t*,12*t*-18:2 synthesis *in vivo* was only detected when cells were grown aerobically (Fig. S1), we propose that UfaD is a newly discovered member of a family of fatty acid desaturases that use O_2_ as an electron acceptor (25). In addition, the failure to observe conversion of unbranched 18:1 to 18:2 *in vitro* suggests that UfaD has a substrate preference for the methyl-branched substrate 11Me-12*t*-18:1, possibly relating to the strength of the C–H bond that must be broken to proceed with the desaturation reaction. The *R. palustris* homologue of UfaD, RPA2571, catalyzes the same reaction *in vivo*, suggesting that the UfaD protein family and its role in FuFA synthesis is conserved in other bacteria. Further information on the specificity for acyl chain and desaturation site, and the requirement for O_2_ and other cofactor(s) for UfaD activity, requires purification and detailed biochemical characterization of this enzyme.

### O_2_ is the source of the oxygen atom in 9M5-FuFA

FuFAs, such as 9M5-FuFA, are one of the few classes of natural fatty acids with an oxygen atom within the hydrophobic portion of the acyl chain. Hydroxylases, lipooxygenases, or other enzymes have been proposed or shown to incorporate an oxygen atom into the furan ring of terpenoids, antimicrobials, and other compounds (14, 22, 30–32). The source of the oxygen atom in the FuFA rings is O_2_; however, the enzyme(s) involved is unknown (33, 34).

Homologs of cytochrome P450 monooxygenase act on fatty acid substrates, and fatty acyl monooxygenases are known to be involved in acyl chain metabolism (35). This work demonstrates that O_2_ is the source of the oxygen atom in 9M5-FuFA and that of UfaO is necessary and sufficient for the synthesis of this FuFA from 11Me-10*t*,12*t*-18:2. This report identifies a potential new class of fatty acyl monooxygenases, UfaO, that use methylated polyunsaturated fatty acids as substrates and directly incorporates O_2_ to form FuFAs. A related protein from *R. palustris* (RPA2570) is required for, and catalyzes, the same step in FuFA synthesis, making it the second reported member of the UfaO protein family.

UfaO homologs are found in many bacteria (24) and are not yet annotated or reported with an enzymatic function. However, they contain a domain of unknown function, DUF1365, that is broadly distributed in Bacteria and Eukarya (36). Thus, it is possible that additional members of the DUF1365 protein family are also heretofore uncharacterized UfaO homologues, or that DUF1365 has a presently unknown role in oxidation reactions. Monoxygenases typically require a reductant for catalysis, and the one used by UfaO is currently unknown. Synthesis of 9M5-FuFA from 11Me-10*t*,12*t*-18:2 and O_2_ did not require the addition of an exogenous reductant, suggesting that sufficient levels of a potential reductant were present in the wheat germ extracts used to assay this reaction *in vitro*. Details on the cofactor(s) and the molecular mechanism of this newly identified FuFA biosynthesis enzyme awaits the progress of ongoing efforts to purify and characterize UfaO proteins that are active with 11Me-10*t*,12*t*-18:2 as a substrate.

### The dimethyl-FuFA, 9D5-FuFA is synthesized from 9M5-FuFA

We also report on the presence and biosynthesis of the dimethyl-FuFA, 9D5-FuFA, in the α-proteobacterium, *R. palustris*. We show that 9D5-FuFA is synthesized from 9M5-FuFA in both the native host and in another bacterium that is engineered to contain the needed enzymes, and we identify FufM (RPA0924) as a SAM-dependent methylase *in vitro* and *in vivo*. When active His_6_-FufM enzyme preparations are incubated in the presence of 18:1(11*Z*) fatty acids, no significant methylation of this substrate is detected, illustrating the relative specificity of FufM for methylating 9M5-FuFA. Comparing the acyl chain specificity of FufM with that of other SAM-dependent fatty acyl methylases (UfaM, cyclopropane fatty acid synthase, etc.) provides an opportunity to understand the molecular basis for hydrocarbon substrate specificity. This work also provides methods and strains to determine whether other FufM homologs produce dimethyl-FuFAs.

### Conclusion

In sum, this work reports new information on the biosynthetic pathway and intermediates in the synthesis of two FuFAs (9M5-FuFA and 9D5-FuFA) and gene products (UfaD, UfaO, and FufM) involved in the production of these FuFAs. Both mono- and dimethyl-FuFAs are synthesized from 18:1 fatty acyl chains on phospholipids, and 9D5-FuFA is synthesized directly from 9M5-FuFA by a previously undescribed SAM-dependent fatty acid methylase, FufM. Homologues of the enzymes involved in monomethyl FuFA synthesis are present across the bacterial phylogeny (24) and in eukaryotes, so it is possible that many other organisms use similar chemical intermediates in a previously undescribed biosynthetic pathway to produce FuFAs. In contrast, FufM homologs are found only in a subset of the organisms that are predicted to contain UfaM, UfaD, and UfaO homologues, suggesting that 9D5-FuFA synthesis is not as widespread as 9M5-FuFA production. The knowledge gained from these studies helps us to understand the chemical transitions needed for FuFA biosynthesis. It also provides the information needed to test for cofactors needed for synthesis of FuFAs and to begin the biochemical analysis of the enzymes that act in this biosynthetic pathway. Further, our findings provide biochemical approaches to overproduce FuFAs for industrial use and understand their reported roles as membrane second messengers and antioxidants (18, 21).

## Experimental procedures

### Bacterial strains and growth

*Escherichia coli*, *R. sphaeroides*, and *R. palustris* strains were grown as described (18, 37) with the exception that when *R. palustris* was grown for phospholipid analysis, it was grown in pMACY-SCAV medium, which replaces yeast extract with 5 g/liter casamino acids (37). When necessary, media were supplemented with 20 µg/ml gentamycin, 50 µg/ml kanamycin, 20 µg/ml chloramphenicol, or 10% sucrose (w/v).

### Gene cloning

Mutant strains and complementation plasmids (Table 1) were made using methods described previously (21, 49) and by using the NEBuilder HiFi DNA Assembly cloning kit (New England Biolabs) (Table S1). Mini-prep kits for cloning were from Zymogen; maxi-prep kits were from Omnigene. See Table S1.

**Table 1:** **Strains and plasmids**

	Relevant genotype	Source/Reference	
**Strains**		
*E. coli*		
DH5α	*supE44 lacu169*(Ф80 *lacZ M15*) *hsdR178 recA1 endA1 gyrA96 thi-1 relA-1*	Ref. 38	
5-alpha	F′/*endA1 hsdR17 (rK^–^ mK^+^) glnV44 thi-1 recA1 gyrA* (Nal^R^) *relA1* Δ*(lacIZYA-argF)U169 deoR (*φ*80dlac*Δ*(lacZ)M15)*	New England Biolabs	
S17-1	C600::RP-4 2-(Tc::Mu) (Kn::Tn7) *thi pro hsdR Hsd* M^+^*recA*	Ref. 39	
B834	F^-^ *ompT hsdS*_B_(r_B_^-^ m_B_^-^) *gal dcm met* (DE3)	Refs. 40–42	
*R. sphaeroides*		
2.4.1	WT	Ref. 43	
Δ*chrR*	*chrR*::*drf*	Ref. 44	
ΔRSP2144	RSP2144::Ω Sm^r^Sp^r^	Ref. 21	
Δ*RSP1087*/ Δ*chrR*	Markerless in-frame deletion of both *RSP1087* and Δc*hrR* using homologous recombination of pKΔ1087mob	This study	
Δ*RSP1088*/ Δc*hrR*	Markerless in-frame deletion of both *RSP1088* and Δc*hrR* using homologous recombination of pKΔ1088mob	This study	
Δ*RSP1089*/Δc*hrR*	Markerless in-frame deletion of both *RSP1089* and Δc*hrR* using homologous recombination of pKΔ1089mob	This study	
Δ*ufaO*/Δc*hrR*	Markerless in-frame deletion of both *RSP1090* and Δc*hrR* using homologous recombination of pKΔ1090mob	This study	
Δ*ufaD*/Δc*hrR*	Markerless in-frame deletion of both *RSP1091* and Δc*hrR* using homologous recombination of pKΔ1091mob	Ref. 18	
ΔufaM/Δ1091/ Δ1090	Markerless in-frame deletion of *RSP2144, RSP1091*, and *RSP1090* using homologous recombination of pKΔ9190mob	This study	
*R*. *palustris*		
CGA009	WT	Ref. 37	
CGA009 Δ*RPA2569*	Markerless in-frame deletion of RPA2569 (*ufaM*) using homologous recombination of pKΔ2569mob	This study	
CGA009 Δ*RPA2570*	Markerless in-frame deletion of RPA2570 (*ufaO*) using homologous recombination of pKΔ2570mob	This study	
CGA009 Δ*RPA2571*	Markerless in-frame deletion of RPA2571 (*ufaD*) using homologous recombination of pKΔ2571mob	This study	
CGA009 Δ*RPA0923*	Markerless in-frame deletion of RPA0923 using homologous recombination of pKΔ0923mob	This study	
CGA009 Δ*RPA0924*	Markerless in-frame deletion of RPA0924 (*fufM*) using homologous recombination of pKΔ0924mob	This study	
CGA009 ΔΔ*RPA0924*/*RPA0923*	Markerless in-frame deletion of RPA0923 and RPA0924 using homologous recombination of pKΔ2324mob	This study	
CGA009 ΔRPA3082	Markerless in-frame deletion of RPA3082 using homologous recombination of pKΔ3082mob	This study	
**Plasmids**		
pK18mobsacB	*oriV oriT mob sacB* Kn^r^	Ref. 45	
pRSBY1	6.6-kb fragment containing *RSP1091-RSP1087* operon	Ref. 21	
pKΔ1087mob	Into the XbaI/HindIII site of pK18mobsacB −1057 bp upstream and +1068 bp downstream of *RSP1087* while deleting 723 bp (of 738 bp) in *RSP1087*. Deletes 241 of the 245 amino acids of *RSP1087*.	This study	
pKΔ1088mob	Into the XbaI/HindIII site of pK18mobsacB −1043 bp upstream and +1104 bp downstream of *RSP1088* while deleting 510 bp (of 561 bp) in *RSP1088*. Deletes 170 of the 186 amino acids of *RSP1088*.	This study	
pKΔ1089mob	Into the XbaI/HindIII site of pK18mobsacB −1066 bp upstream and +1104 bp downstream of *RSP1089* while deleting 1161 bp (of 1206 bp) in *RSP1089*. Deletes 387 of the 402 amino acids of *RSP1089*.	This study	
pKΔ1090mob	Into the XbaI/HindIII site of pK18mobsacB −1032 bp upstream and +1002 bp downstream of *RSP1090* stop while deleting 708 bp (of 750 bp) in *RSP1090*. Deletes 212 of the 250 amino acids of *RSP1089*. Potential promoter of *RSP1089* is kept.	This study	
pKΔ9190mob	Into the XbaI/HindIII site of pK18mobsacB −745 bp upstream *RSP1091* and +1265 bp downstream of *RSP1090* stop while deleting 708 bp (of 750 bp) in *RSP1090*. Deletes all amino acids of 1091 and 1090.	This study	
pKΔ2569mob	−1012 bp upstream and +1026 bp downstream of *RPA2569* was inserted into upstream flanking base 3696, downstream flanking base 3951 of pK18mobsacB. This deleted the whole coding sequence of *RPA2569*.	This study	
pKΔ2570mob	−1030 bp upstream and +1024 bp downstream of *RPA2570* was inserted into upstream flanking base 3696, downstream flanking base 3951 of pK18mobsacB. This deleted the whole coding sequence of *RPA2570*.	This study	
pKΔ2571mob	−1034 bp upstream and +1048 bp downstream of *RPA2571* was inserted into upstream flanking base 3696, downstream flanking base 3951 of pK18mobsacB. This deleted the whole coding sequence of *RPA2571*.	This study	
pΔ0923mob	−1030 bp upstream and +1030 bp downstream of *RPA0923* was inserted into upstream flanking base 3696, downstream flanking base 3951 of pK18mobsacB. This deleted the whole coding sequence of *RPA0923*.	This study	
pΔ0924mob	−1030 bp upstream and +1032 bp downstream of *RPA0924* was inserted into upstream flanking base 3696, downstream flanking base 3951 of pK18mobsacB. This deleted the whole coding sequence of *RPA0924*.	This study	
pΔ2324mob	−1530 bp upstream *RPA0923* and +1530 bp downstream of *RPA0924* was inserted into upstream flanking base 3696, downstream flanking base 3951 of pK18mobsacB. This deleted the whole coding sequence of *RPA0923* and *RPA0924*.	This study	
pΔ3082mob	−1025 bp upstream and +1000 bp downstream of *RPA3082* was inserted into upstream flanking base 3696, downstream flanking base 3951 of pK18mobsacB. This deleted the whole coding sequence of *RPA3082*.	This study	
pBBR1MCS-5	Broad host range cloning vector, Gm^R^, *lacZ*α, *mob*, *mod, rep*	Ref. 46	
pBBR2144	*RSP2144* interrupts the *lacZ*α gene in pBBR1MCS-5 to remove the BamHI site (upstream flanking base is 1518, downstream flanking base is 1519).	This study	
pBBR2569	*RPA2569* replaces the *lacZ*α gene in pBBR1MCS-5 (upstream flanking base is 3012, downstream flanking base is 3379).	This study	
pBBR2570	*RPA2570* replaces the *lacZ*α gene in pBBR1MCS-5 (upstream flanking base is 3012, downstream flanking base is 3379).	This study	
pBBR2571	*RPA2571* replaces the *lacZ*α gene in pBBR1MCS-5 (upstream flanking base is 3012, downstream flanking base is 3379).	This study	
pBBR0924	*RPA0924* replaces the *lacZ*α gene in pBBR1MCS-5 (upstream flanking base is 3012, downstream flanking base is 3379).	This study	
pIND5	pIND4 NcoI site replaced with NdeI site, Kn^r^.	Ref. 21	
pRL59187	*RSP1091-1087* containing its native promoter −22, was amplified from pRSBY1 and ligated into the XbaI/HindIII site of pIND5.	This study	
pRL101	*ufa*M in pIND5	Ref. 18	
p5U9187	*ufa*M coding sequence was amplified from pRL101 and ligated into the XbaI site of pRL59187.	This study	
p5U9190	Around the world PCR was done with p5U9187 to delete *RSP1087*, *RSP0188*, and *RSP1089* to yield a construct with *ufa*M, *RSP1091*, and *RSP1090*.	This study	
p5RPA2571-2568	Coding sequence of CGA009 *RPA2571*, *RPA2570*, *RPA2569*, *RPA2568* inserted in the NdeI-cut site of pIND5.	This study	
pVP302K	His_8_ expression vector, Kn^r^	Ref. 47	
pVP1091his	Coding sequence of *RSP1091* in pVP302K creating an N-terminal His_8_-tag-RSP1091.	This study	
pRARE	Plasmid encoding rarely used tRNAs: AGG, AGA, AUA, CUA, CCC, GGA.	Novagen	
pEU-His-TEV-eGFP		Ref. 48
pEU90his	Coding region of *RSP1090* replacing eGFP in pEU-His_6_-TEV-eGFP.	This study
pEU91his	Coding region of *RSP1091* replacing eGFP in pEU- His_6_-TEV-eGFP.	This study
pEU0924his	Coding region of *RPA0924* replacing eGFP in pEU- His_6_-TEV-eGFP.	This study

### FAME analysis

Samples were prepared and analyzed as described previously (18) with the exceptions that they were kept away from light to minimize fatty acid photooxidation and photoisomerization, all solutions were degassed with N_2_, and FAMEs were resuspended in 250 µl of hexane for analysis by GC-MS.

### Hydrogenation and identification of unknown FAME

FAMEs were created as described previously (18) from 13 ml of aerobically grown Δ*chrR*Δ*ufaO* cells and dried down under N_2_. This material was resuspended in 3 ml of dichloromethane in a round-bottom flask, degassed with H_2_ prior to the addition of 2 mg of 5% palladium on activated charcoal (Sigma), and stirred. The sample was sparged with H_2_ in a balloon and incubated for 2 h at room temperature and processed as described above for GC-MS analysis. Preparation of the DMOX (4,4-dimethyloxazoline) derivatives was as described (50) with the exception that the reactions were incubated at 190 °C for 18 h; extracted by adding 5 ml of diethyl ether/hexane (1:1), 5 ml of water, and ∼0.3 g of NaCl; and dried with sodium sulfate and N_2_. The products were reconstituted in 100 µl hexane, and 1-µl aliquots (split 1:10) were analyzed on a Thermo Scientific Q Exactive GC Orbitrap GC-MS/MS system equipped with a Trace 1310 GC and TriPlus RSH autosampler using the following oven program: 150 °C isothermal for 4 min, 6 °C/min ramp to 245 °C, and then 80 °C/min ramp to 325 °C and isothermal at 325 °C for 2 min. The MS transfer line was heated to 300 °C, the MS source was held at 250 °C, and the ionization energy was 70 eV. The GC inlet temperature was set to 275 °C, analyzer mass resolution was set to 70,000, and the automatic gain control target and maximum ion times were set to automatic. Spectral interpretation and elucidation of the location of double bonds by the mass spectra of DMOX derivatives is described in (51–56) (Christie, W. W. (2017) Mass Spectrometry of Dimethyloxazoline and Pyrrolidine Derivatives Epoxy, Furanoid and Alkoxy Fatty Acids; https://www.lipidhome.co.uk/ms/dmox/dmox-epoxy/index.htm; Accessed July 13, 2017).

### Isolation of ΔchrRΔufaO phospholipids and FAME creation for NMR analysis

FAMEs were created as described above from 1.6 × 10^12^ Δ*chrR*Δ*ufaO* cells. The crude mixture of FAMEs was purified using HPLC. Briefly, the mixture of FAMEs was dissolved in acetonitrile (2 ml) and filtered through a 0.2-µm PTFE syringe filter. The sample was then injected onto a Shimadzu LC-20 equipped with a Phemonenex Luna 5-µm C18(2), 100 Å, 250 × 10-mm column held at 50 °C and a photodiode array detector (Shimadzu, SPD-M20). The mobile phase was a binary gradient of acetonitrile and water that ramped from 20% acetonitrile to 100% acetonitrile over 35 min. Fractions were collected by hand and were selected based on the absorption chromatogram at λ = 300 nm. Each fraction was dried under nitrogen, and FAME identity and purity was determined using an Agilent 7890A/5975C GC-MS as described previously (18).

### Ectopic expression of FuFA biosynthetic genes

Plasmid pIND5 was used for expression in *R. sphaeroides* and pBBR1mcs5 for expression in *R. palustris*. Triplicate biological cultures were separately treated with 1 mm IPTG (for *R. sphaeroides*) for 3 h before preparing FAMEs (see above) from samples containing equivalent cell numbers.

### Purification of His_8_-UfaD protein

A 500-ml culture of log-phase B834 *E. coli* cells (40, 41), containing pVPufaD, was inoculated into autoinducing ZMS-80155 (57) medium containing plasmid pRARE2 (Novagen) to induce expression of *N*-terminally octahistidine-tagged protein (His_8_-UfaD), with shaking for ∼24 h at room temperature (58). The cells were harvested by centrifugation, and the resulting pellet was resuspended in lysis buffer (50 mm Na_2_HPO_4_, pH 7.5, 1 m NaCl, 20 mm imidazole, 10% Sodium Lauroyl Sarcosinate, and 1× Halt protease inhibitor (Pierce)), sonicated on ice, pulsing every 30 s, for 20 min, and centrifuged for 1 h at 20,000 × *g*. The resulting supernatant was incubated with 4 ml of Ni-nitrilotriacetic acid–agarose resin (Fisher/Pierce) for 30 min at 4 °C, poured into a column, and then washed with 50 ml of wash buffer I (50 mm Na_2_HPO_4_, pH 7.5, 1 m NaCl, 35 mm imidazole) and 50 ml of wash buffer I containing 50 mm imidazole. Protein was removed with 16 ml of elution buffer (50 mm Na_2_HPO_4_, pH 7.5, 1 m NaCl, 500 mm imidazole). Fractions containing the most protein were combined and concentrated using a YM10 centrifugal filter (Millipore) and dialyzed into dialysis buffer (50 mm HEPES, pH 7.5, 20 mm NaCl). All protein preparations were done fresh for each assay. Protein concentration was estimated using the Bradford assay (Bio-Rad and Pierce).

### In vitro assay of His_8_-UfaD activity

The phospholipid substrate was purified from a Δ*ufaD* strain, and a phospholipid micelle solution (in water) was created (59) and quantitated by a lipid phosphorous assay (60). The enzyme reaction contained ∼3 mm phospholipid and 0.7 µm His_8_-UfaD protein in 20 mm potassium phosphate buffer, pH 7.4, 0.5 mg/ml BSA and was incubated at 30 °C overnight.

### Cell-free synthesis and in vitro assay of His_6_-UfaO and His_6_-FufM activity

pEU-ufaO was generated by cloning the *ufaO* coding region into pEU to produce an N-terminally hexahistidine-tagged protein (His_6_-ufaO and His_6_-FufM). Synthesis of both UfaO and FufM was achieved in a wheat germ cell-free extract using published translation conditions (61). The fatty acid modification reaction was run concurrently with the translation reaction by adding ∼3 mm phospholipid substrate, prepared as described above, to the reaction. The phospholipid substrate was purified from a Δ*ufaO* strain (for the 9M5-FuFA synthesis assay) and a Δ*chrR* strain (for the FufM assay) to generate phospholipid lysosomes containing putative enzyme substrates and quantified as above (51). As determined by SDS-PAGE imaging with a Bio-Rad stain-free imaging system (61), the UfaO reaction contained ∼60 µm UfaO; however, FufM concentration could not be calculated this way as it ran concurrently with other proteins in the wheat germ cell-free system.

### ^18^O_2_ modification of fatty acid chains

Cell-free protein synthesis was performed in the presence of ^18^O_2_ (99% isotopic enrichment, from Sigma–Aldrich, 602892-1L) in a sealed reaction tube prepared in an anaerobic hood or in air. Cultures were incubated in a sealed container, and a balloon with ^18^O_2_, N_2_, or compressed air was added to each culture. Data shown are representative results from duplicate technical replicates of each experiment.

## Data availability

All data are contained within this article and the supporting information.

## Supplementary Material

Supporting Information
