# Supplementary material for: A bacterial biosynthetic pathway for methylated furan fatty acids
Source: J Biol Chem. 2020 May 20;295(29):9786–801. doi: 10.1074/jbc.RA120.013697 (PMC7380195; doi:10.1074/jbc.RA120.013697)
Supplement: Supporting Information [file supp_295_29_9786__index.html]

A bacterial biosynthetic pathway for methylated furan fatty acids — Methylated Furan Fatty Acid Biosynthesis — A bacterial biosynthetic pathway for methylated furan fatty acids — EDITORS' PICK: Methylated furan fatty acid biosynthesis — Supporting Information 

# A bacterial biosynthetic pathway for methylated furan fatty acids

## Supporting Information

- Supporting Information (to be published online) - Formated PDF of primer table and supplementary figures.
