# Supplementary material for: A bacterial biosynthetic pathway for methylated furan fatty acids
Source: J Biol Chem. 2020 May 20;295(29):9786–801. doi: 10.1074/jbc.RA120.013697 (PMC7380195; doi:10.1074/jbc.RA120.013697)
Supplement: Supporting Information [file supp_RA120.013697_159884_2_supp_529390_qz8jzd.pdf]

SUPPORTING INFORMATION

A biosynthetic pathway for methylated furan fatty acids

Rachelle A.S. Lemke[1,2,3], Stephanie M. Olson[1,2,3], Kaitlin Morse[2,3], Steven D. Karlen[2,3,4], Alan Higbee[2,3,5], Emily T. Beebe[3], John Ralph[2,3,4], Joshua J. Coon[3,5,6], Brian G. Fox[2,3,4], and Timothy J. Donohue[1,2,3,]*

From the [1]Department of Bacteriology, [2]Wisconsin Energy Institute, [3]Great Lakes Bioenergy Research Center, [4]Department of Biochemistry, [5]Genome Center of Wisconsin, [6]Department of Biomolecular Chemistry, University of Wisconsin-Madison, Madison, Wisconsin 53706

Running Title: Methylated Furan Fatty Acid Biosynthesis

[*]To whom correspondence should be addressed: Dept. of Bacteriology, University of Wisconsin, Madison, WI 53706. Tel.:608-262-4663; E-mail: tdonohue@bact.wisc.edu

Keywords: fatty acid, fatty acid metabolism, fatty acyl methylase, fatty acid modification, lipid metabolism, oxygenated fatty acids, phospholipid, polyunsaturated fatty acid

Supplementary Table 1. Primers

Plasmid	**Primer**
pKΔ1087	Vector pK18*mobsac*B primers:<br>pk18HiFi-For 5′ - AAGCTTGGCACTGGCCGTCG - 3′<br>pK18HiFi-Rev 5′ - TCTAGAGGATCCCCGGGTACCGAGC - 3′<br><br>Deletes 241 of the 244 amino acids in RSP1087.<br><br>Upstream RSP1087 primers: 1057 bp upstream of RSP1087<br>Up-1087-For 5′ - CGGGGATCCTCTAGACGGGGTCCGAGGGGCCGC - 3′<br>Up-1087-Rev 5′ - TCTCAGCCGTTCATCTCTCGATCCGTCTCAGTGTCGCCAC - 3′<br><br>Downstream RSP1087 primers: 1068 bp downstream of RSP1087<br>1087Down-For 5′ - GAGATGAACGGCTGAGACGGACGCGCC - 3′<br>1087Down-Rev 5′ - GCCAGTGCCAAGCTTCGAACGAGGAACGGGTCGG - 3′
pKΔ1088	Vector pK18*mobsac*B primers: as listed above<br>Deletes 170 of 185 amino acids in RSP1088<br><br>Upstream RSP1088 primers: 1066 bp upstream of RSP1088<br>Up-1088-For 5′ - CGGGGATCCTCTAGATGGGCTGGCTGGCCGAGG - 3′<br>Up-1088-Rev 5′ TCGGCCACCGAACTCACGGGTCAACTCTCCGG - 3′<br><br>Downstream RSP1089 primers: 1104 bp downstream of RSP1088<br>1088Down-For 5′ - TGAGTTCGGTGGCCGAGCTTGTGGCG - 3′<br>1088Down-Rev 5′ - GCCAGTGCCAAGCTTCTGCGGGTGATCGGAGAC - 3′
pKΔ1089	Vector pK18*mobsac*B primers: as listed above<br>Deletes 387 of the 400 amino acids in RSP1089<br><br>Upstream RSP1089 primers: 1066 bp upstream of RSP1089<br>Up-1089-For 5′ - CGGGGATCCTCTAGACATCACCTACTGGATGAACTCGCTG - 3′<br>Up-1089-Rev 5′ - AGCAGGAGCGCACTCACCGCGTGACG - 3′<br><br>Downstream RSP1089 primers: 1127 bp downstream of RSP1089<br>1089Down-For 5′ - TGAGTGCGCTCCTGCTCCTTGCCACC - 3′<br>1089Down-Rev 5′ - GCCAGTGCCAAGCTTCCGTGTCGCGCAGATCGG - 3′

pKΔ1090	Vector pK18*mobsac*B primers: as listed above<br>Deletes 212 of the 250 amino acids in RSP1090<br><br>Upstream RSP1090 primers: 1016 bp upstream of RSP1060<br>90Xba-For 5′ - GTGGTCTAGAGTGACATGAGCTTCGGCGC - 3′<br>90Hind3-Rev 5′ - GAGCGCaAGCTTCGAGGCGAAGGACC - 3′<br><br>Downstream RSP1090 primers: 1002 bp downstream of RSP1090<br>del90-UP1 primer 5′ - GCGGCCTGAGGGGCGCGTC - 3′<br>del90-Down1 primer 5′ - CGCAGCCGCCCCGCGC - 3′
pKΔΔ91-90	Vector pK18*mobsac*B primers: as listed above<br>Deletes all 683 amino acids of RSP1091 and RSP1090<br><br>Upstream RSP1091 primers: 745 bp upstream RSP1091<br>HiFi-Up1091-For 5′ - CGGGGATCCTCTAGAGCGCAGACGGTCCAGCGC - 3′<br>HiFi-Up1091-Rev 5′ - GCACTCACGCCAATTTCCCCGTTCCCTTGC - 3′<br><br>Downstream RSP1090 primers: 1265bp downstream of RSP1090<br>HiFi-1090Down-For 5′ - GAAATTGGCGTGAGTGCGCGGCTTCTG - 3′<br>HiFi-1090Down-Rev 5′ - GCCAGTGCCAAGCTTGTAAGGGGGATGAGGGCG - 3′
pKΔ2569	pK18msB-For 5′ - AAAGTGCGTCGGGTGATG - 3′<br>pK18msB_Rev 5′ - TGTTTCCAGTCGGTAGATATTCCACAAAACAGC - 3′<br><br>Upstream RPA2569<br>US2569-For 5′ - CGCAGCAGGATACTGTTCCATTGCTTGCGC - 3′<br>US2569-Rev 5′ - AGCATCACCCGACGCACTTTGTTCCATGAGGACGGGCTTC - 3′<br><br>Dowstream RPA2569<br>DS2569-For 5′ - ATATCTACCGACTGGAAACACGCACAGGATGGTCACGATG - 3′<br>DS2569-Rev 5′ - TGGAACAGTATCCTGCTGCGGTCTTGCC - 3′
pKΔ2570	pK18msB-ForA 5′ - AAAGTGCGTCGGGTGATGC - 3′<br>pK18msB-RevA 5′ - TGTTTCCAGTCGGTAGATATTCCAC - 3′<br><br>Upstream RPA2570<br>up2570-For 5′ - TACTTTAGCTGCTATTCGGCCGCTTCGC - 3′<br>up2570-Rev 5′ - AGCATCACCCGACGCACTTTGACGGTCTGTTCGCGCAG - 3′<br><br>Dowstream RPA2570<br>dn2570-For 5′ - ATATCTACCGACTGGAAACAATGAAATCGACCTCTCGCC - 3′<br>dn2570-Rev 5′ - GCCGAATAGCAGCTAAAGTATGAGCCCGGC - 3′
pKΔ2571	pK18msB-For 5′ - AAAGTGCGTCGGGTGATG - 3′<br>pK18msB-Rev 5′ - TGTTTCCAGTCGGTAGATATTCCACAAAACAGC - 3′<br><br>Upstream RPA2571<br>up2571-For 5′ - GAGGGCCATGTCCAAGCCCCCTCTAGCC - 3′<br>up2571-Rev 5′ - AGCATCACCCGACGCACTTTGAGCACCTGGAAATACGCG - 3′<br><br>Dowstream RPA2571<br>dn2571-For 5′ - ATATCTACCGACTGGAAACATTCCGCGCTGCAGCCGCG - 3′<br>dn2571-Rev 5′ - GAGGGGGCTTGGACATGGCCCTCCTCGCCGC - 3′

pKΔ0923	pK18msB-For 5′ - AAAGTGCGTCGGGTGATG - 3′<br>pK18msB-Rev 5′ - TGTTTCCAGTCGGTAGATATTCCACAAAACAGC - 3′<br><br>Upstream RPA0923<br>US0923-For 5′ - ATATCTACCGACTGGAAACACCATCTTCCAGCGTCAGC - 3′<br>US0923-Rev 5′ - GCCCGCTCATCATCCACCCGTCGTTCGAC - 3′<br><br>Dowstream RPA0923<br>DS0923-For 5′ - CGGGTGGATGATGAGCGGGCTCGCCTCG - 3′<br>DS0923-Rev 5′ - AGCATCACCCGACGCACTTTTGACACCCCACTCGTCGC - 3′
pKΔ0924	pK18msB-For 5′ - AAAGTGCGTCGGGTGATG - 3′<br>pK18msB-Rev 5′ - TGTTTCCAGTCGGTAGATATTCCACAAAACAGC - 3′<br><br>Upstream RPA0924<br>US0924-For 5′ - ATATCTACCGACTGGAAACATCTGTCTCCGGTTTGCCG - 3′<br>US0924-Rev 5′ - GCACGAACAGTCATAGCGTGGCGGAGCG - 3′<br><br>Dowstream RPA094<br>DS0924-For 5′ - CACGCTATGACTGTTCGTGCACCGGGGC - 3′<br>DS0924-Rev 5′ - AGCATCACCCGACGCACTTTCTCGCCGAGCAGATCGGC - 3′
pKΔ0923-0924	pK18msB-For 5′ - AAAGTGCGTCGGGTGATG - 3′<br>pK18msB-Rev 5′ - TGTTTCCAGTCGGTAGATATTCCACAAAACAGC - 3′<br><br>Upstream RPA0923/0924<br>UP923_924-For 5′ - ATATCTACCGACTGGAAACAACCCGGTCGCGCTCGCTC - 3′<br>UP923_924-Rev 5′ - GCACGAACAGCATCCACCCGTCGTTCGACCG - 3′<br><br>Dowstream RPA0923/0924<br>DS923_924-For 5′ - CGGGTGGATGCTGTTCGTGCACCGGGGC - 3′<br>DS923_924-Rev 5′ - AGCATCACCCGACGCACTTTGTCCCGAGCTGGACCCGC - 3′
pKΔ3082	pK18msB-For 5′ - AAAGTGCGTCGGGTGATG - 3′<br>pK18msB-Rev 5′ - TGTTTCCAGTCGGTAGATATTCCACAAAACAGC - 3′<br><br>Upstream RPA3082<br>Up3082-For 5′ - ATATCTACCGACTGGAAACACTCAGGCTCAGCAGCCTTC - 3′<br>Up3082-Rev 5′ - TCCACCGGGGCGACTTGCCCCAAAACCG - 3′<br><br>Dowstream RPA3082<br>Dn3082-For 5′ - GGGCAAGTCGCCCCGGTGGAGTGCAACAC- 3′<br>Dn3082-Rev 5′ - AGCATCACCCGACGCACTTTCAGCGACAGCTCGTAGATGG - 3′
pBBR1MCS-5	pBBR1MCS5-For<br>5′ - AGCTGTTTCCTGTGTGAAATTGTTATCCGCTC - 3′<br>pBBR1MCS5-Rev<br>5′ - GCGTTAATATTTTGTTAAAATTCGCGTTAAATTTTTGTTAAATC - 3′
pBBR2569	2569-For-BBR 5′ - GAATTTTAACAAAATATTAACGCATGTCCGAGTTGGTAGCGC - 3′<br>2569-Rev-BBR 5′ - ATTTCACACAGGAAACAGCTTCAGCCCGCCTTTGCGAAC - 3′
pBBR2570	2570-For-BBR 5′ - GAATTTTAACAAAATATTAACGCATGGCCCTCCTCGCCGCC - 3′<br>2570-Rev-BBR 5′ - ATTTCACACAGGAAACAGCTTTAGTGAGTATAAGCATCGTGACCGCCGG - 3′
pBBR2571	2571-For-BBR 5′ - GAATTTTAACAAAATATTAACGCATGCGCGTTGCTATCGTTG - 3′<br>2571-Rev-BBR 5′ - ATTTCACACAGGAAACAGCTCTATTCGGCCGCTTCGCG - 3′

pBBR0924	0924-For-BBR 5′ - GAATTTTAACAAAATATTAACGCATGAGCGGGCTCGCCTCG - 3′<br>0924-Rev-BBR 5′ - ATTTCACACAGGAAACAGCTTCAGGCCGGCTTCAGGCG - 3′
pRL59187	1087-XbaI-For 5′ - TCCGTATCTAGAGCAAGGGAACGGGG - 3′<br>1087-Hind3-rev 5′ - CGCGTAAGCTTCAGCCCTTGC - 3′
p5U9187	UfaMind5-For 5′ - CACACATCTAGAATTAAAGAGGAGAAATTACATATGACGG - 3′<br>ufaMind5-XbaI-REV 5′ - GGGCCGCGTAAGCTCTAGAAA - 3′
p5U9190	Del-5U9190new-For 5′ - CAAGACGTCACGCGGTGA - 3′<br>Del-Endp5u9187-Rev 5′ - GAAGCTTAATTAGCTGAGCTTGGACTC - 3′
p5Rpa2571-2568	RPA-NdeI-2571-For 5′ - GCTTCATATGCGCGTTGCTATCG - 3′<br>RPA2568-HindIII-Rev 5′ - GCCTTGCGGAACATTCCATC - 3′
pVP1091his	pVP302K-For 5′ - AAGCTTCGCCTGGGGTAATG - 3′<br>pVP302K-Rev 5′ - TGCGATCGCGCTCTGAAAATAC - 3′<br>RSP1091-For 5′ - CAGAGCGCGATCGCACCATTCGAGACGTCTGAGTTCGCGCG - 3′<br>RSP1091-Rev 5′ - TTACCCCAGGCGAAGCTTTCAGGCCGCGGCCGAGGC - 3′
pEU90his	pEU-For 5′ - TAGTTTAAACGAATTCGAGCTCGG - 3′<br>pEU-Rev 5′ - GTGATGATGATGATGATGTCCCATTAAC - 3′<br>1090TEV-For 5′ - GACATCATCATCATCATCACGCATTGGCAAGCGAAAATCTG - 3′<br>1090TEV-Rev 5′ - GCTCGAATTCGTTTAAACTATCACCGCGTGACGTCTTG - 3′
pEU0924his	EUhis0924-For 5′ - GACATCATCATCATCATCACAGCGGGCTCGCCTCGCTG - 3′<br>EUhis0924-Rev 5′ - GCTCGAATTCGTTTAAACTATCAGGCCGGCTTCAGGCG - 3′<br>pEUhistev-0924-For 5′ - TAGTTTAAACGAATTCGAGCTCGG - 3′<br>pEUhistev-0924-Rev 5′ - GTGATGATGATGATGATGTCCCATTAAC - 3′

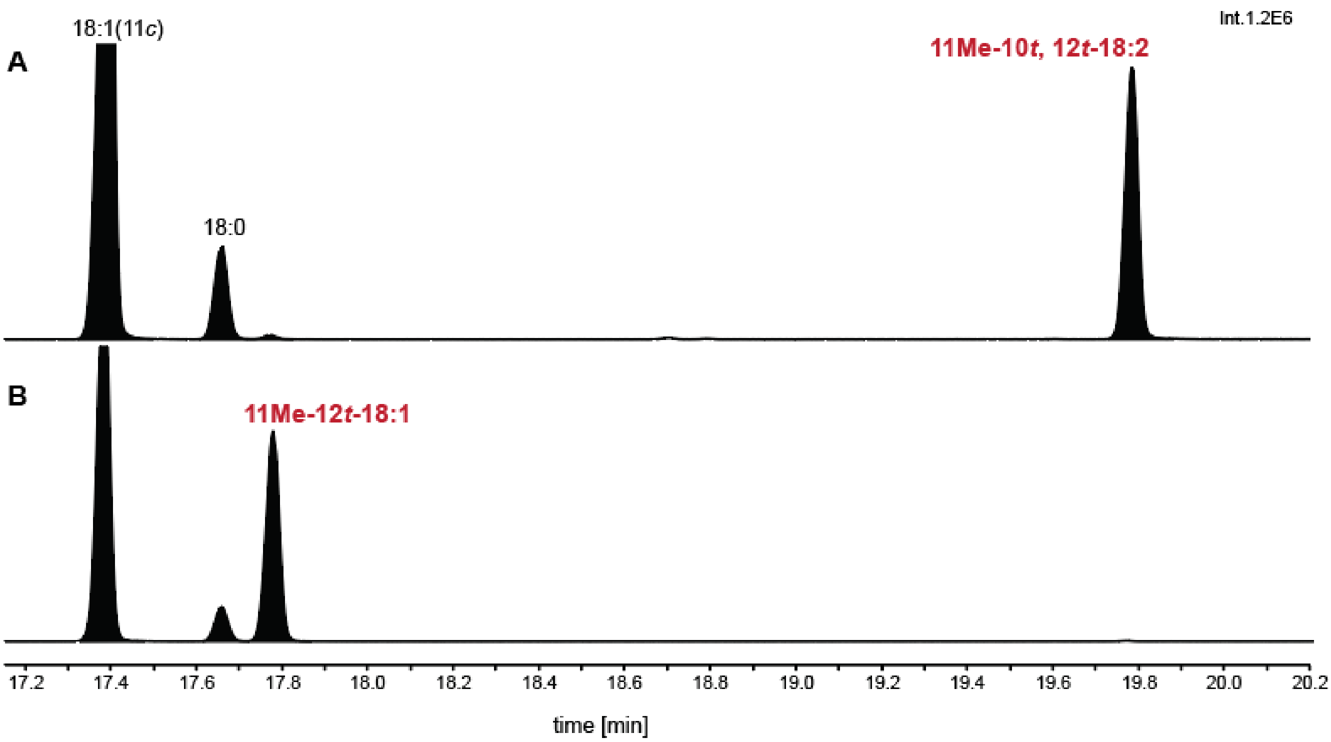

Supplemental Figure S1. O_2 is required to produce 11Me-10*t*, 12*t*-18:2. GC elution profiles of FAMEs from Δ*chrR*Δ*ufaO* cells grown aerobically (panel A) or anaerobically (panel B).

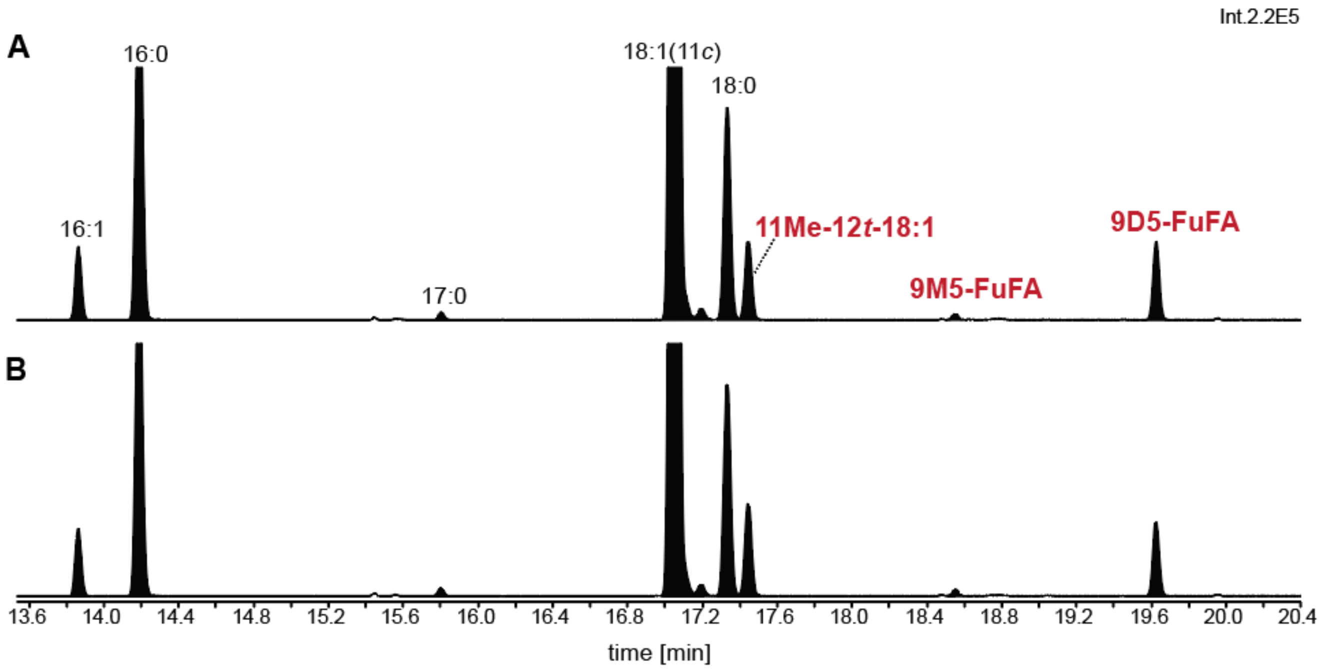

Supplemental Figure S2. *Rs. palustris* RPA3082 is not needed to produce 9M5- or 9D5-FFA. GC elution profiles of FAMEs from wild type *Rs. palustris* CGA009 cells (panel A) and a Δ*RPA3082* mutant (panel B).
